# A human genome-wide loss-of-function screen identifies effective chikungunya antiviral drugs

**DOI:** 10.1038/ncomms11320

**Published:** 2016-05-12

**Authors:** Alexander Karlas, Stefano Berre, Thérèse Couderc, Margus Varjak, Peter Braun, Michael Meyer, Nicolas Gangneux, Liis Karo-Astover, Friderike Weege, Martin Raftery, Günther Schönrich, Uwe Klemm, Anne Wurzlbauer, Franz Bracher, Andres Merits, Thomas F. Meyer, Marc Lecuit

**Affiliations:** 1 Max Planck Institute for Infection Biology, Department of Molecular Biology, Charitéplatz 1, 10117 Berlin, Germany; 2 Steinbeis Innovation gGmbH, Center for Systems Biomedicine, Haydnallee 21, 14612 Falkensee, Germany; 3 Institut Pasteur, Biology of Infection Unit, 28 rue du Dr. Roux, 75015 Paris, France; 4 Inserm U1117, 28 rue du Dr. Roux, 75015 Paris, France; 5 Institute of Technology, University of Tartu, Nooruse 1, 50411 Tartu, Estonia; 6 Institute of Virology, Charité University Medicine, Charitéplatz 1, 10117 Berlin, Germany; 7 Max Planck Institute for Infection Biology, Core Facility Experimental Animals, Charitéplatz 1, 10117 Berlin, Germany; 8 Ludwig-Maximilians-University, Department of Pharmacy-Center for Drug Research, Butenandtstraße 5-13, 81377 Munich, Germany; 9 Paris Descartes University, Sorbonne Paris Cité, Division of Infectious Diseases and Tropical Medicine, Necker-Enfants Malades University Hospital, Institut Imagine, 149, rue de Sèvres 75743 Paris, France

## Abstract

Chikungunya virus (CHIKV) is a globally spreading alphavirus against which there is no commercially available vaccine or therapy. Here we use a genome-wide siRNA screen to identify 156 proviral and 41 antiviral host factors affecting CHIKV replication. We analyse the cellular pathways in which human proviral genes are involved and identify druggable targets. Twenty-one small-molecule inhibitors, some of which are FDA approved, targeting six proviral factors or pathways, have high antiviral activity *in vitro*, with low toxicity. Three identified inhibitors have prophylactic antiviral effects in mouse models of chikungunya infection. Two of them, the calmodulin inhibitor pimozide and the fatty acid synthesis inhibitor TOFA, have a therapeutic effect *in vivo* when combined. These results demonstrate the value of loss-of-function screening and pathway analysis for the rational identification of small molecules with therapeutic potential and pave the way for the development of new, host-directed, antiviral agents.

## Abstract (web summary)

Chikungunya virus is a mosquito transmitted untreatable emergent pathogen that causes joint pain and fever. Here the authors perform a host genome-wide loss-of-function screen to identify targets for chikungunya antiviral drugs and validate hits using a mouse model of chikungunya infection.

## Graphical abstract

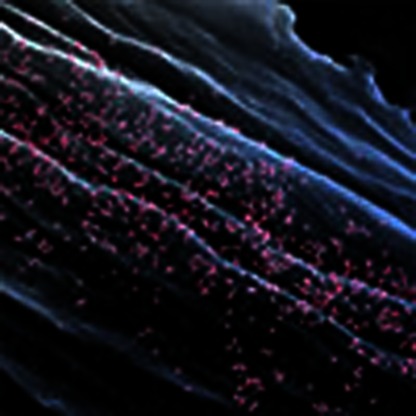

Chikungunya virus (CHIKV) is a mosquito-borne alphavirus that causes chikungunya fever, an acute infection characterized by arthralgia and frequently complicated by chronic joint symptoms^1^. Originating from Africa, CHIKV has spread to Asia, emerged in Southern Europe and reached the Americas^1^. Despite its severe health and economic impact, little is known about the cell biology of CHIKV infection and, as for all emerging infections, neither prophylactic nor therapeutic strategies are available on the market^1^. In the absence of available therapeutics and vaccines, there is an urgent need for antimicrobial agents to combat emerging infections^2^. As illustrated by the latest generation of drugs available to treat human immunodeficiency virus (HIV)-infected or hepatitis C virus (HCV)-infected patients, deciphering that the biology of infections has proven successful for the development of highly effective drugs targeting different viral proteins^3^,^4^. However, this virus-directed strategy for drug discovery is time-consuming and not rapidly applicable to emerged pathogens^2^. In the context of emerging infections, targeting host factors critical to infection is a promising approach^5^,^6^,^7^. Indeed, this host-directed antiviral design can be coupled with a drug repositioning strategy^8^ that could lead to the rapid identification of antiviral agents.

RNA interference (RNAi)-mediated loss-of-function screens have enabled the identification of novel host factors and pathways that are either important for replication (proviral) or limit replication (antiviral) of different viruses^9^,^10^,^11^,^12^,^13^,^14^, and therefore represent potential target for therapeutic purposes. Here we perform a host genome-wide loss-of-function screen to identify the host factors implicated in CHIKV replication in human cells. We analyse the cellular pathways and factors necessary for CHIKV infection and identify antiviral drugs with both *in vitro* and *in vivo* efficacy.

## Results

### Identification of CHIKV pro- and anti-viral factors

**Figure 1:**
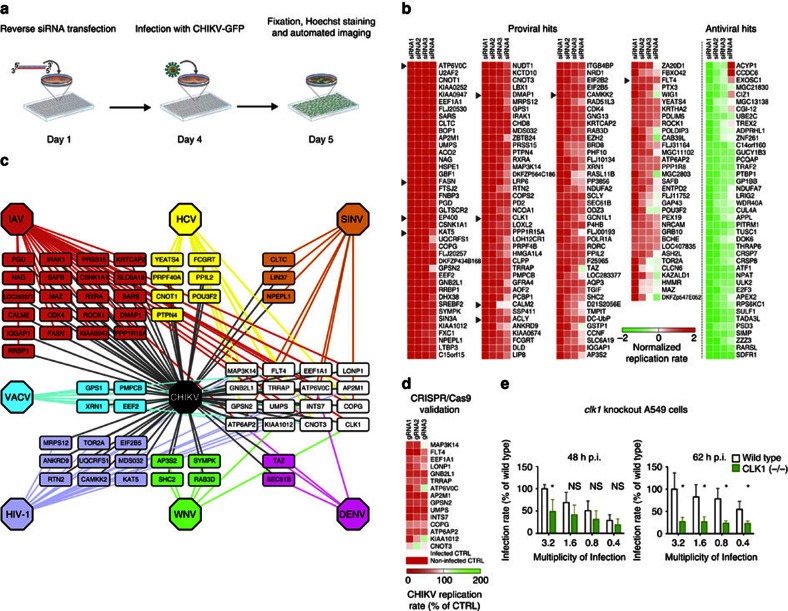
Primary screen for CHIKV host cell factors. (**a**) Outline of screening procedure. (**b**) Heatmap of all identified proviral and antiviral hits showing replication data (*Z*-scores) of the four most efficient siRNAs. Arrowheads indicate genes experimentally characterized in this study. (**c**) Requirement of identified proviral factors for other viruses based on published loss-of-function studies. Viruses other than CHIKV are indicated by specific colours, and colour-coded boxes contain genes shared between CHIKV and the corresponding virus. White boxes contain genes shared between CHIKV and more than one other virus. Full details in Supplementary Data 3. (**d**) Heatmap illustrating the replication capability of CHIKV in Cas9-positive HEK-293T cells expressing the indicated gRNAs. Cells were infected with CHIK-GFP at MOI 6 for 24 h (*n*=9). (**e**) Validation of CLK1 as CHIKV relevant host factor. Infection rate of A549 cells depleted for CLK1 by CRISPR/Cas9 (see Supplementary Fig. 1j for more details) and infected with CHIKV-GFP for the indicated periods of time (*n*=9 for each data set). Data represent the means±s.e.m. of three independent experiments and were analysed using one-way analysis of variance with Tukey's post test. (*P<0.05; ^NS^*P*≥0.05). CTRL, control; NS, not significant.

A host genome-wide loss-of-function screen was performed by reversely transfecting a genome-wide library of ∼60,000 short interfering RNAs (siRNAs) targeting ∼17,000 annotated and ∼6,000 predicted genes into CHIKV-permissive human cells (HEK-293) for 3 days. Transfected cells were infected with a recombinant CHIKV-expressing green fluorescent protein (GFP) (CHIKV-GFP) in infected cells. At 18 h post infection (p.i.), cells were fixed and analysed by automatic fluorescence microscopy to quantify the ratio of infected to uninfected cells (Fig. 1a). To reduce the number of false-positive hits, 7,561 non-expressed genes (Supplementary Fig. 1a) and 3,274 toxic siRNAs (Supplementary Fig. 1b,c) were excluded following transcriptional microarray and viability analyses, respectively. RNAi data were then analysed statistically using cellHTS2 (ref. 15), and normalized using as positive control, an siRNA targeting the viral E1 gene, and as negative control AllStars siRNA (Supplementary Fig. 1d). A *Z*-score transformation was applied to center and scale the plate-wise data (Supplementary Fig. 1d), and the medians of resulting values of at least three independent replicates were used for redundant siRNA activity (RSA) analysis^16^. Using RSA log *P* values of ≤−2 as cutoff, we identified 279 proviral and 269 antiviral factors (Supplementary Fig. 1e,f). To minimize off-target false positives, we further validated these hits with three additional siRNAs per gene. On the basis of the calculated cSSMD (collective strictly standardized mean difference) values, which reflect effect strength and significance^17^ (Supplementary Fig. 1g,h), we validated 156 proviral and 41 antiviral host factors (Fig. 1b; Supplementary Data 1 and 2). Strikingly, 66 of these proviral genes have been identified in previous screens with influenza A virus (IAV)^9^,^14^, HCV^18^,^19^, sindbis virus (SINV)^10^,^11^, dengue virus (DENV)^20^, West Nile virus (WNV)^21^, HIV-1 (refs 13, 22) or vaccinia virus (VACV)^23^ (Fig. 1c; see Supplementary Data 3 for details and references). Moreover, 16 factors are relevant for multiple viruses (white boxes in Fig. 1c), therefore representing the central biological nodes for broad-spectrum antiviral therapeutic intervention.

**Figure 2:**
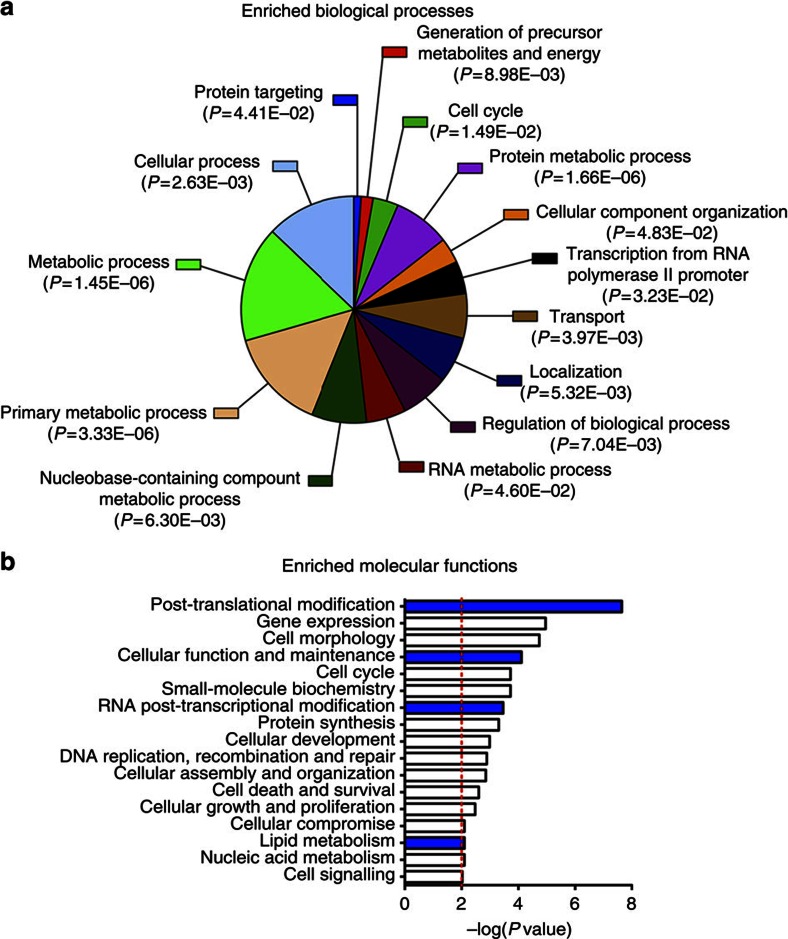
Screening results analysis. (**a**,**b**) PANTHER (http://www.pantherdb.org/) and Ingenuity (www.ingenuity.com) gene ontology (GO) analysis for enrichment of certain categories in the context of biological processes or molecular functions, respectively. See Supplementary Fig. 2 for a graphical illustration of some pathways included in the GO terms labelled in blue in **b**.

To definitely validate these 16 factors, we used an independent method, the CRISPR/Cas9 technology, to generate cells deficient for each of these genes. We observed a significant reduction of CHIKV replication with guide RNAs (gRNA)-transduced Cas9-expressing cells for 14 out of 16 genes, establishing that they are proviral factors (Fig. 1d,e; Supplementary Fig. 1i,j; Supplementary Data 4) and highlighting the strong reliability of the RNAi-validated hits of our study. Among the proviral factors, the three largest protein–protein interaction networks are involved in transcription, translation and signalling, respectively (Supplementary Fig. 1k). Gene enrichment analysis revealed biological processes (Fig. 2a) and molecular functions (Fig. 2b) required for infection, including cell metabolism, post-transcriptional and post-translational modifications, and cellular function and maintenance (Fig. 2a,b; Supplementary Fig. 2a–d). Genes associated with lipid metabolism, in particular fatty acid synthesis, were statistically overrepresented within the hits (Fig. 2b; Supplementary Fig. 2c).

### Fatty acid synthesis is required for CHIKV replication

**Figure 3:**
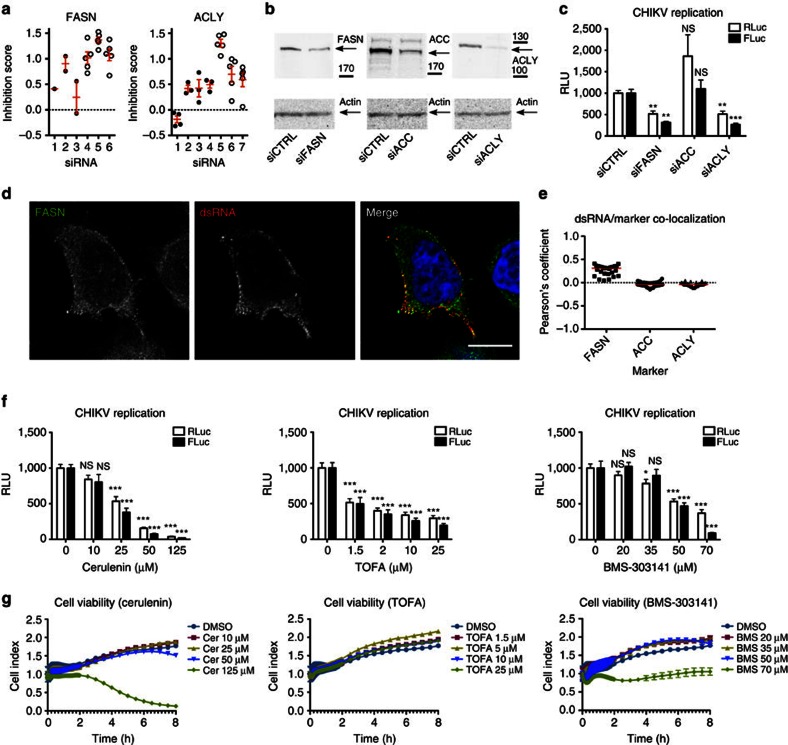
Fatty acid synthesis requirement for CHIKV life cycle. (**a**) Impact of FASN or ACLY knockdown on CHIKV replication. Closed and open symbols indicate replicates from the primary screen and during validation, respectively. (**b**) Western blot showing silencing efficiency of siRNAs used in **c**. (**c**) Impact of FASN-, ACC- and ACLY-specific siRNAs on CHIKV replication (*n*=10 for each data set). (**d**) Confocal section of CHIKV replicon-infected HeLa cells labelled for FASN, dsRNA and 4,6-diamidino-2-phenylindole (DAPI; blue). Scale bar, 10 μm. (**e**) Co-localization analysis of cells labelled as in **d** and in Supplementary Fig. 3b, plotted as Pearson's coefficient per cell. Each symbol corresponds to a cell stack from three independent experiments (*n*=29 cells for FASN, 30 cells for ACC and 31 cells for ACLY); median values shown in red. (**f**) Effect of FASN (cerulenin, *n*=12 for each data set), ACC (TOFA, *n*=11 for each data set) and ACLY (BMS-303141 *n*=11 for each data set) inhibitors on CHIKV replication. (**g**) Real-time cell toxicity assay performed on HeLa cells (*n*=3 for each point). Excepted for **b** and **d** where representative images are shown and for **g** where the mean±s.d. is shown for each point of a representative experiment, all data represent the means±s.e.m. of three independent experiments analysed using one-way analysis of variance with Tukey's post test (*P<0.05; **P<0.01; ***P<0.001; ^NS^*P*≥0.05). NS, not significant.

Cytosolic fatty acid synthesis involves the following three enzymes: (i) ATP citrate lyase (ACLY), needed for the formation of acetyl CoA, (ii) acetyl CoA carboxylase (ACC), which transforms acetyl CoA into malonyl CoA, and (iii) fatty acid synthase (FASN), which converts acetyl and malonyl CoA into palmitate (Supplementary Fig. 2c). Both FASN and ACLY were unambiguous hits (Figs 1b and 3a; Supplementary Fig. 2c). Interestingly, FASN activity is required for the replication of other enveloped viruses^24^,^25^ and both the enzymes are present in late replication complexes (Rc) of a closely related alphavirus (Semliki Forest virus)^26^. To analyse the functional implication of lipid metabolism in CHIKV replication, we used a CHIKV replicon, allowing measurement of genomic and subgenomic viral RNA (vRNA) expression simultaneously (Supplementary Fig. 3a). Consistent with the screening results, the expression of both the vRNA forms was impaired in HeLa cells depleted in either FASN or ACLY, but not in cells depleted in ACC (Fig. 3b,c). Immunofluorescence analysis further showed that Rc co-localized with FASN (Fig. 3d,e; Supplementary Fig. 3b). We next independently confirmed these results pharmacologically. Infected HeLa cells were exposed to cerulenin (FASN inhibitor), 5-tetradecyloxy-2-furoic acid (TOFA, ACC inhibitor), BMS-303141 (ACLY inhibitor) or vehicle only (dimethylsulphoxide, DMSO). All three inhibitors induced a significant and dose-dependent reduction in genomic and subgenomic vRNA synthesis (Fig. 3f), with no deleterious effect on cell viability (Fig. 3g). Together, these results show that fatty acid synthesis is necessary for CHIKV vRNA replication.

### Rational identification of antiviral drugs

On the basis of the demonstration of the antiviral activity of all lipid biosynthesis inhibitors targeting the proviral factors identified by the host genome-wide loss-of-function screen, we next considered all the other hits as putative drug targets. Therefore, to exploit the translational potential of our screen, we systematically assessed the antiviral activity of chemical compounds targeting the identified proviral factors involved in CHIKV replication. We interrogated specialized databases (www.drugbank.ca^27^; lincs.hms.harvard.edu/kinomescan^28^; www.ebi.ac.uk/chembl^29^), linking several drugs to their experimentally proven target proteins/genes, and identified 52 compounds interacting with the gene products of 14 distinct CHIKV proviral genes. In addition, we generated three compounds against the CDC-like kinase 1 (CLK1), a proviral factor for CHIKV, IAV and WNV (Fig. 1c; Supplementary Data 3; Supplementary Methods). Of these, 20 compounds—8 of them already Food and Drug Administration approved—interacting with six distinct proviral factors or pathways (Table 1) potently inhibited CHIKV replication with no significant toxicity (Table 1; Supplementary Fig. 4; Supplementary Data 5). These antiviral compounds inhibit the following targets and pathways: (i) the vacuolar-type H+ ATPase (vATPase); (ii) CLK1; (iii) the fms-related tyrosine kinase 4 (FLT4 or VEGFR3); (iv) calmodulin signalling; (v) fatty acid synthesis; (vi) the K (lysine) acetyltransferase 5 (KAT5 or TIP60; see Supplementary Data 6 for more details).

**Figure 4:**
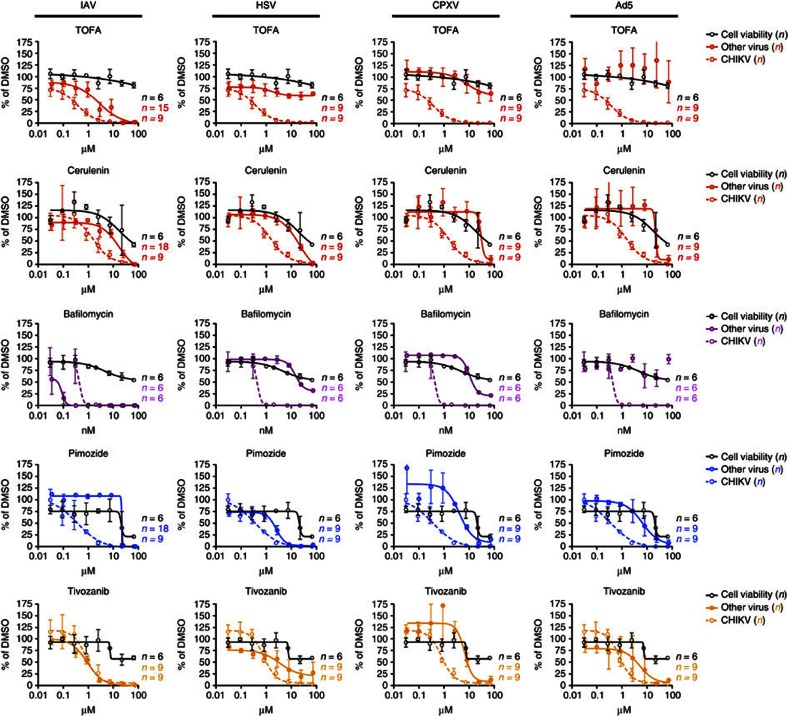
Effect of the identified antiviral drugs on different classes of viruses. Dose–response curves (coloured solid lines) performed on HEK-293 cells or A549 cells (in case of IAV infection), pretreated for 2 h with the indicated drugs and then infected with distant classes of viruses. Black solid lines and coloured dashed lines indicate the corresponding dose–response curves determined for cell viability and CHIKV infection, respectively (Table 1; Supplementary Fig. 4). Data were obtained from the combination of at least two independent experiments (*n* is indicated in each panel) and are depicted as mean±s.e.m. for each point.

Since five of these six druggable hits were also identified as proviral factors in other RNAi screens (Fig. 1c; Supplementary Data 3), we sought to characterize the efficacy of inhibitors of these hits on the replication of a wide spectrum of viral species. Cells were treated with specific inhibitors of fatty acid synthesis, calmodulin signalling, FLT4 and vATPase, and infected with cytosolic double-stranded DNA virus (cowpox, CPXV), nuclear double-stranded DNA virus (herpes simplex type 1, HSV-1, or adenovirus type 5, Ad5) or negative-stranded segmented RNA virus (IAV) (Fig. 4). Interestingly, differences in the antiviral activity of these compounds were observed between viral species, suggesting that the identified cellular pathways are required for the replication of several, but not all virus types, and that the antiviral effect observed for CHIKV is genuine and not the consequence of a general toxic effect on cell metabolism induced by the inhibitors.

### *In vitro* characterization of the antiviral compounds

**Figure 5:**
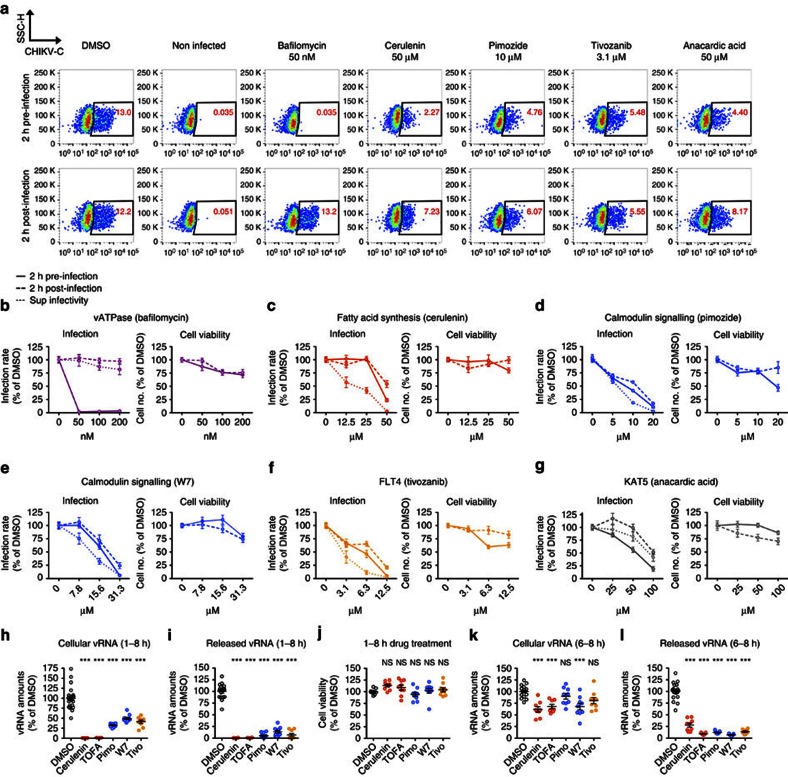
*In vitro* validation of selected chemical inhibitors and proviral factors. (**a**–**g**) Infection rate and cell viability for compounds specific to vATPase (bafilomycin, *n*=12, 6, 12 and 6 for all conditions at increasing concentrations), calmodulin (pimozide, *n*=12, 12, 12 and 9 for all conditions at increasing concentrations and W7, *n*=9 for all data sets), fatty acid synthesis (cerulenin, *n*=9, 6, 6 and 9 for all conditions at increasing concentrations), FLT4 (tivozanib, *n*=12, 6, 9 and 12 for all conditions at increasing concentrations) and KAT5 (anacardic acid, *n*=9 for all data sets) administrated to CHIKV-infected HEK-293T cells as indicated. Cells were treated 2 h before (solid lines) or 2 h after (dashed lines) infection with CHIKV C21 (MOI 40) and left until 8 h p.i. to avoid multiple cycles of infection. Viral infectivity and cell viability were then measured by flow cytometry after intracellular staining of CHIKV capsid (CHIKV-C, see **a** for an example). Equal amounts of supernatants from the 2 h post treatment conditions were measured for viral infectivity on Vero cells (dotted lines) to detect eventual defects in viral release. (**h**,**i**) Quantitative PCR with reverse transcription quantification of cell- (*n*=20, 9, 9, 12, 12 and 9 in displayed order) (**h**) and supernatant-associated (*n*=18, 6, 6, 12, 12 and 9 in displayed order) (**i**) vRNA performed on RNA extracts of HeLa cells infected with CHIKV C21 (MOI: 50) for 1 h and treated with cerulenin (50 μM), TOFA (25 μM), pimozide (pimo, 10 μM), W7 (20 μM), tivozanib (tivo, 5 μM) or vehicle alone for an additional 7 h. (**j**) Cell viability measured at the end of the experiment shown in **h**, using the CellTiter-Glo kit (*n*=9 for all data sets). (**k**,**l**) Cell- and supernatant-associated vRNA measured on CHIKV-infected HeLa cells treated with the indicated drugs from 6 to 8 h p.i. (*n*=18, 9, 8, 9, 9 and 9 for *m* and *n*=18, 9, 9, 9, 9 and 9 for *n* in displayed order). Data represent mean+s.e.m. of at least three independent experiments, analysed by one-way analysis of variance with Tukey's post test (***P<0.001; ^NS^*P*≥0.05).

To get some insight into the mechanism of action of the identified antiviral compounds, we investigated which stage of the viral life cycle was affected by inhibition of each of these six distinct druggable targets. In a first set of experiments, single-cycle CHIKV infection was measured before or after viral entry (Fig. 5a). Infectivity of the supernatants of post-entry-treated cells was also measured to detect the possible defects on late-stage CHIKV life cycle. As expected for the control of vacuolar pH by vATPase^30^, its inhibitor bafilomycin specifically blocked CHIKV entry (Fig. 5b). In contrast, the drugs targeting the other proviral hits all affected the CHIKV cycle post entry (Fig. 5c–g). This was also true for the specific calmodulin inhibitor (W7), included in the experiment as a specificity control for this pathway (Fig. 5e). Because of the incomplete inhibition observed with the CLK1 inhibitor KH-CB19 at early times p.i., we were not able to precisely determine the step of infection controlled by CLK1 (Supplementary Fig. 5a). Importantly, consistent with the mechanism of action of the FLT4 inhibitors that are known to suppress FLT4 phosphorylation, using validated specific antibodies (Supplementary Fig. 5b), we observed increased FLT4 phosphorylation in cells infected with CHKV (Supplementary Fig. 5c).

One of the druggable targets (KAT5) is part of the acetylation complex Tip60-EP400, recently described as a general antiviral factor^31^. Since, somewhat surprisingly, several components of this complex were identified as proviral factors in our screen (Supplementary Fig. 2a), we further investigated this target using three siRNAs against different subunits (Supplementary Fig. 5d) in HeLa and HEK-293T cells. We observed that the pro- or anti-viral contribution of the Tip60-EP400 complex was cell line specific for both CHIKV and SINV (Supplementary Fig. 5e), and therefore did not pursue this lead.

We next characterized the step in CHIKV replication cycle targeted by the specific inhibitors of fatty acid synthesis, calmodulin signalling and FLT4 that act after entry. Infected HeLa cells were exposed to cerulenin, TOFA, pimozide, W7, tivozanib or vehicle only (DMSO). All the tested inhibitors significantly reduced vRNA synthesis (Fig. 5h) and strongly inhibited viral release in the supernatants (Fig. 5i), with no detectable cell toxicity (Fig. 5j) when applied following 1-h infection with wild-type CHIKV isolate C21. Of note, a delayed treatment (6–8 h p.i.) had only a minor impact on vRNA replication but induced a major defect in viral release (Fig. 5k,l), suggesting that the pathways targeted by these inhibitors are also involved in the late phase of CHIKV life cycle.

### *In vivo* characterization of the antiviral compounds

**Figure 6:**
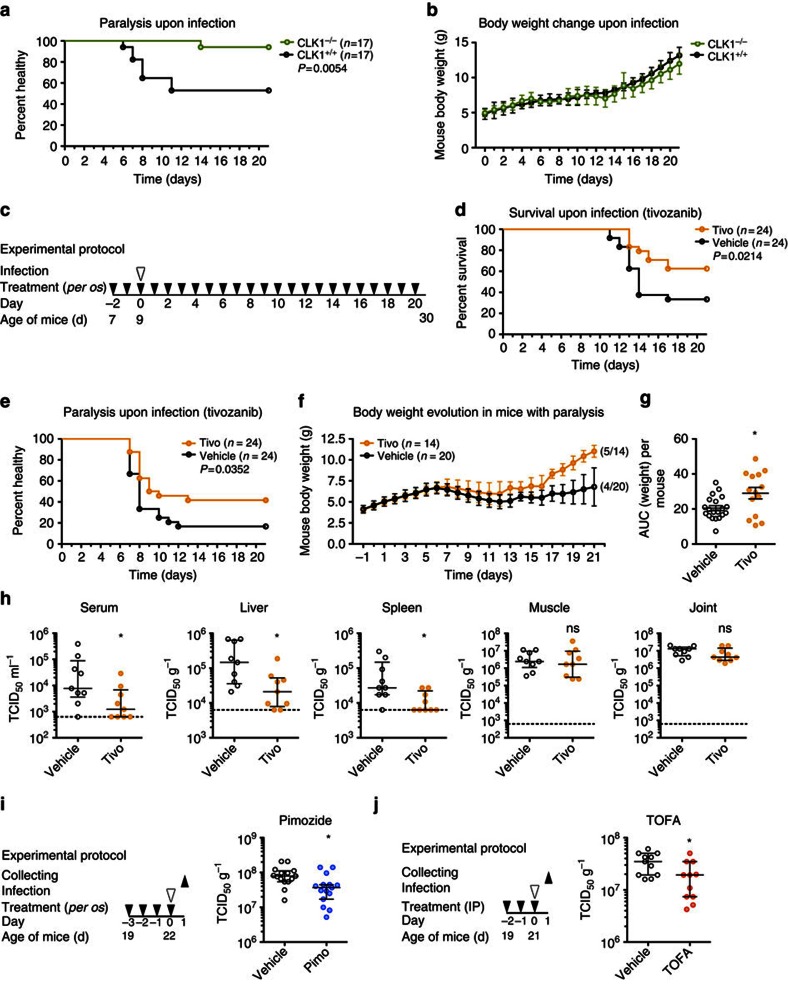
*In vivo* validation of selected chemical inhibitors and proviral factors. (**a**,**b**) Health status and body weight evolution in 9-day-old C57BL/6^*clk1*−/−^ or C57BL/6^*clk1*+/+^ mice infected intradermally with CHIKV C21 (10^4^ PFU) and killed at the appearance of paralysis. (**c**) Experimental design of the intradermal infection of the young mouse model used for tivozanib. (**d**–**f**) Effect of daily tivozanib (tivo) treatment on C57BL/6 mouse survival, paralysis and body weight change in response to CHIKV C21 infection. (**g**) Health status of each mouse with paralysis, estimated by measuring the area under the body weight curve. (**h**) CHIKV viral load measured 3 days post infection in the indicated organs obtained from mice treated with tivozanib as in **c** (*n*=9 for all data sets). (**i**,**j**) Experimental design of the footpad infection of adult mice model used and viral titres measured in C57BL/6 mice treated with pimozide (pimo, *per os*, *n*=15 for both data sets) or TOFA (i.p., *n*=11 for both data sets) or the corresponding vehicles before infection with CHIKV C21 (10^3^ PFU). Data in **b**,**f** and **g** represented as the mean±s.e.m.; in **h**,**i** and **j** as the median±interquartile range; each dot represents one mouse. All data obtained from at least two independent experiments. Statistics were calculated using Log-rank (Mantel–Cox) test in **a**,**d** and **e**, two-sided *t*-test for two independent samples in **g** and Mann–Whitney test in **h**,**i** and **j**, (*P<0.05; ^NS^*P*≥0.05). AUC, area under curve; d, days; i.p., intraperitoneal; NS, not significant.

We next aimed at translating these *in vitro* results *in vivo*. We therefore generated C57BL/6^*clk1*−/−^ mice (see Methods) and investigated the requirement for CLK1 in CHIKV *in vivo* infection^32^. Weight-matched 9-day-old C57BL/6^*clk1*−/−^ mice developed significantly less paralysis (∼50%) than control isogenic mice (5.8%; *P*=0.005, log-rank test; Fig. 6a,b), identifying CLK1 as a valid *in vivo* target for antiviral drug development. To analyse the antiviral effect of tivozanib (targeting FLT4) *in vivo*, 7-day-old C57BL/6 mice were treated with the drug or vehicle only for 2 days and infected with CHIKV at day 9. Tivozanib administration was continued for 21 days (Fig. 6c). Tivozanib caused a significant reduction in mortality of CHIKV-infected mice (Fig. 6d; *P*=0.02, log-rank test), protected from the onset of paralysis (Fig. 6e; *P*=0.03, log-rank test) and had a positive impact on body weight gain (Fig. 6f,g; *P*=0.0133, two-sided *t*-test). Tivozanib treatment also significantly reduced CHIKV viral load in different vital organs (Fig. 6h). Finally, we tested the *in vivo* efficacy of the calmodulin inhibitor pimozide and the fatty acid synthesis inhibitor TOFA, which have both been used previously for therapeutic purposes in mice^33^,^34^,^35^. These drugs had a detectable impact on mouse growth and were therefore not used further in neonatal mice, but tested in the adult mouse footpad injection model^36^,^37^,^38^. Mice were pretreated for at least 3 days, infected and assessed 18 h p.i. CHIKV titres in the footpad revealed that both the drugs significantly reduced replication (Fig. 6i,j), indicating that fatty acid synthesis and calmodulin also constitute promising targets for antiviral purposes.

### Effect of drug combination *in vitro* and *in vivo*

**Figure 7:**
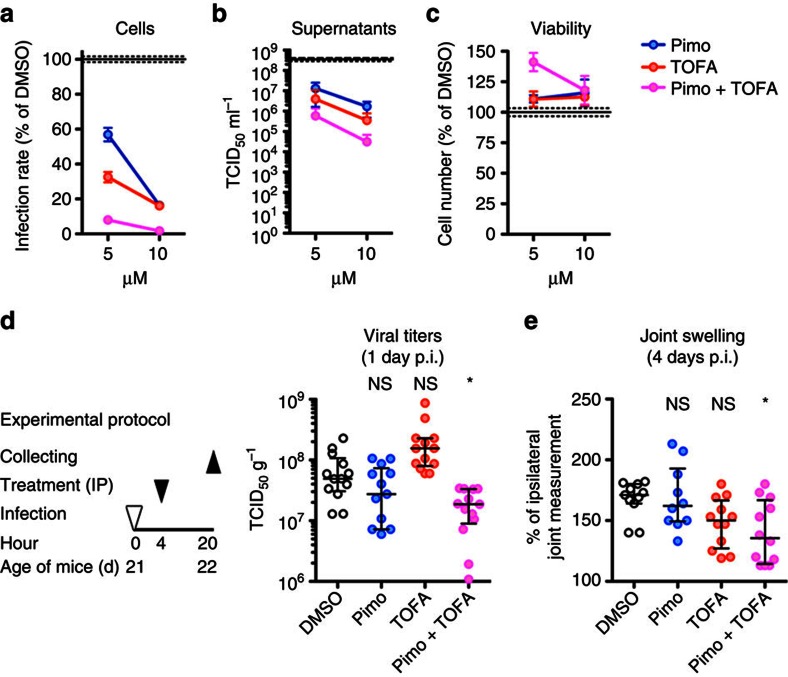
Impact of pimozide and TOFA combination on CHIKV replication *in vitro* and *in vivo*. (**a**–**c**) Infection rate (*n*=9 for all data sets at increasing concentrations; *n*=51 for DMSO) (**a**), supernatant titration (*n*=8 for all data sets at increasing concentrations; *n*=57 for DMSO) (**b**) and cell viability (**c**) of HEK-293T cells infected with CHIKV-GFP (MOI: 0.5) for 1 h, and then exposed to the indicated concentrations of antiviral drugs for 23 h. Solid and dashed black lines indicate the mean±s.e.m. of the DMSO control (pimo=pimozide). Data in **a** and **c** were measured by flow cytometry. (**d**) Experimental design of the footpad CHIKV infection model of adult mice used to measure the therapeutic effect on CHIKV replication of pimozide, TOFA or their combination (*n*=13 for all data set, one outlier for pimo is not shown in the graph but was considered for statistics). (**e**) Footpad swelling measured 4 days post infection in mice treated as in **d** (*n*=11, 10, 12 and 12 in displayed order). The experiment shown in **e** was repeated twice. All the other data were obtained from at least three independent experiments. Data in **a**,**b** and **c** are expressed as the mean±s.e.m. and in **d** and **e** are shown as the median±interquartile range; each dot represents one mouse. Statistics were calculated using the Kruskal–Wallis test with the Dunn's post test (*P<0.05; ^NS^*P*≥0.05) NS, not significant.

Having identified and characterized compounds with anti-CHIKV activity both *in vivo* and *in vitro*, we combined drugs to improve antiviral activity. We focused on pimozide and TOFA, since they both demonstrated an antiviral effect in the same mouse model of CHIKV infection. We initially tested the effect of drugs administrated in combination on HEK-293T cells infected with CHIKV for 1 h. In comparison with monotherapy, TOFA and pimozide combination resulted in increased inhibition of CHIKV infection rate (5 μM each, interaction effect=0.44; lower=0.36; upper=0.54; *P*<0.001, two-factor design method) and CHIKV release (5 μM each, interaction effect=3.44; lower=0.68; upper=17.41; *P*=0.133, two-factor design method), with no deleterious effect on cell viability (Fig. 7a–c). We then evaluated the anti-CHIKV effect of drug combination *in vivo*, in adult mice therapeutically treated with pimozide and TOFA alone or in combination. Importantly, pimozide and TOFA combination caused a significant reduction in CHIKV replication (Fig. 7d) (interaction effect=0.16; lower=0.05; upper=0.47; *P*<0.001, two-factor design method) and in CHIKV-induced joint swelling (Fig. 7e) (interaction effect=0.94; lower=0.79; upper=1.13; *P*=0.506, two-factor design method), when therapeutically administrated to infected adult mice, suggesting that combining different drugs identified in the RNAi screen increases their antiviral effect.

## Discussion

Despite major outbreaks over the past decade in Africa, the Indian Ocean and Asia and its recent emergence in the Americas, still little is known regarding CHIKV cell biology. CHIKV infection in human is associated with acute and chronic symptoms, which pathophysiology remains incompletely understood, and there is no commercially available vaccine or drugs to tackle this global public health issue. To better characterize CHIKV interaction with human cells, we performed a genome-wide RNAi screen and report here on the identification of 156 cellular factors required for CHIKV life cycle and 46 antiviral factors. Importantly, follow-up studies of those hits led to the identification of molecular pathways and cellular functions required for multiple steps of CHIKV life cycle, from viral entry, replication to viral release. For example, we demonstrated the critical role of the vacuolar ATPase in viral entry, and lipid biosynthesis in vRNA replication and release. Therefore, our study represents a major advance in the field of CHIVK cell biology, and will pave the way for detailed mechanistic studies for each of the pro- and anti-CHIKV host factors we have identified, similarly to other loss-of-function screens published for other human viruses^9^,^10^,^11^,^13^,^14^,^18^,^19^,^20^,^21^,^22^,^23^.

We followed an innovative translational approach by directly exploiting the results of the genome-wide loss-of-function screen to identify druggable pathways and corresponding antiviral compounds. Indeed, using a drug repositioning strategy, we identified a series of chemical inhibitors, directed against five druggable proviral hits, with *in vitro* and *in vivo* antiviral activity, either alone or in combination. Since some of the drugs are already Food and Drug Administration approved for other diseases, this has the potential to considerably shorten the time-consuming and expensive ‘hit-to-lead' and ‘lead-optimization' phases, making our approach of particular interest for antiviral drug discovery in the field of emerging infectious diseases. Importantly, most of the identified proviral hits and all druggable targets we followed up are also critical for the replication of other very diverse viral species (including IAV, SINV, HIV-1 and HCV; see Fig. 1c), highlighting the relevance of this strategy for the identification of broad-spectrum antiviral compounds and the potential for broadly active, host-directed, antiviral therapies (for example, pimozide that had an antiviral effect both for CHIKV and HSV-1 in Fig. 4).

This proof-of-concept study, based on a genome-wide loss-of-function screen, led to the identification of antiviral targets, pathways and compounds for which antiviral efficacy was proven *in vitro*, as well as *in vivo*, although it did not completely block viral replication in this setting. Additional studies will be required for the selection of the best target inhibitors and lead optimization, before clinical development. Since our approach led to the selection of a number of independent targets and pathways, and for each of those to the identification of multiple antiviral compounds, pre-clinical studies may exploit the potential synergistic effect of drug combination. With this in mind, we showed that the combination of two drugs targeting independent cellular pathways (TOFA and pimozide) have an increased activity when combined *in vitro* as well as *in vivo*, where it exhibits a therapeutic antiviral effect.

In conclusion, we have demonstrated here the significance of host genome-wide loss-of-function screens for the rational identification of antiviral targets and their accelerated *in vitro* and *in vivo* validation for emerging pathogens, for which therapeutics and vaccines are critically missing^1^,^2^.

## Methods

### Cells and viruses

The HEK-293 cells (CRL-1573, ATCC-LGC) used for the genome-wide screening, the drug screening and the drugs testing on different viral species were cultured in DMEM (Life Technologies 31966-047) supplemented with 4 mM L-glutamine, 100 U ml^−1^ penicillin/streptomycin and 10% FCS. The HEK-293T cells (CRL-3216, ATCC-LGC) used for the CRISPR/Cas9 validation were cultured in DMEM (Life Technologies 31966-047) supplemented with 4 mM L-glutamine, 100 U ml^−1^ penicillin/streptomycin and 10% FCS. The HEK-293T cells (CRL-3216, ATCC-LGC) and HeLa cells used for follow-up studies were cultured in DMEM (high glucose, GlutaMAX, pyruvate) supplemented with 100 U ml^−1^ penicillin/streptomycin (Life Technologies 15140-122) and 10% FCS. Human lung epithelial A549 cells (CCL-185, ATCC-LGC) were cultured in DMEM supplemented with 4 mM L-glutamine, 1 mM sodium pyruvate, 100 U ml^−1^ penicillin/streptomycin and 10% FCS. Vero cells (CCL-81, ATCC-LGC) were cultured in DMEM, supplemented with 100 U ml^−1^ penicillin/streptomycin and 5% FCS for cell passaging and 2% FCS for viral titration with the TCID50 assay. C6/36 cells (CRL-1573, ATCC-LGC) were cultured in Leibovitz's L-15 medium (Life Technologies 11415-049) supplemented with 10% FCS, 0.01 g ml^−1^ tryptose phosphate broth and 1 × minimum essential media (MEM) non-essential amino acids (Life Technologies 11140-050). All cell lines were maintained at 37 °C in the presence of 5% CO_2_ with the exception of C6/36 that were cultured at 28 °C with no CO_2_.

The following viruses were used: CHIKV-GFP^39^ (a kind gift of S. Higgs, Kansas, USA), CHIKV C21 and SINV AR339 (both kind gifts of P. Desprès, Paris, France). Viral production and titration were performed as previously detailed^40^. Briefly, viruses were expanded on C6-36 cells and virus stocks were stored at −80 °C before titration by plaque assay on Vero cells. CHIKV-GFP was concentrated using Vivaspin concentrators (molecular weight cut off (MWCO) 100,000 kDa, Sartorius). Viral titration of cell supernatants (Fig. 7b) was performed by standard TCID50 assay on Vero cells using eight serial dilutions (1/10 steps).

A CHIKV replicon vector, expressing Renilla luciferase (Rluc) fused with nsP3 and firefly luciferase (Ffluc) under the subgenomic promoter (see Supplementary Fig. 3a), was used for experiments in Fig. 3c,f. For immunofluorescence experiments in Fig. 3d,e and Supplementary Fig. 3b, cells were infected with a CHIKV replicon vector expressing only Fluc under the subgenomic promoter. Chikungunya viral replicon particles (VRPs) were produced using the split-helper system as described previously^41^. In short, three different vRNAs were produced *in vitro* (mMESSAGE mMACHINE SP6 Kit (Ambion)) from separate plasmids: replicon RNA (including the 5′ and 3′ CHIKV replication signals but lacking the structural genes) and two different helper RNAs for the expression of capsid protein and envelope proteins. Three transcribed RNAs were electroporated into BHK-21 cells and kept at 32 °C for 72 h before collecting the media to collect VRPs. Indirect immunofluorescence microscopy was used to titre VRPs.

GFP-expressing recombinant viruses used in Fig. 4 were cowpox virus strain Brighton Red vBRFseR (CPXV; provided by K. Tischer, Freie Universität Berlin, Germany), herpes simplex virus 1 strain KOS K26GFP (HSV-1; provided by P. Desai, John Hopkins University, USA) and Adenovirus serotype 5 expressing GFP from CMV immediate-early promoter (Ad5). Virus stocks were grown in Vero E6 cells (HSV-1, CPXV) or HEK-293 cells (Ad5) for 5–10 days before being collected, resuspended in PBS and lysed by three sequential freeze–thaws. After removal of cellular debris by centrifugation, stocks were frozen at −80 °C until use. Highly pure stocks of adenovirus were generated by two sequential CsCl centrifugation steps followed by dialysis and freezing at −80 °C until use. Viral titre of the frozen stocks was determined by both GFP expression at 1 day post infection and plaque formation at 7 days post infection of Vero E6 or HEK-293 cells. Virus stocks were >98% pure for GFP expression. The IAV/WSN/1933(H1N1) (provided by St. Jude Children's Research Hospital, USA) was produced by reverse genetics as described^42^, subsequently propagated in the allantoic cavaties of 9- or 10-day-old embryonated chicken eggs and used for experimentation.

### siRNA screening

HEK-293 cells were used for the screening because of their concomitant permissiveness to CHIKV infection and to high-efficiency siRNA transfection. Transfections of siRNAs in a 384-well plate format were carried out as recently described^9^. Briefly, all siRNAs (Qiagen Hu_Genome 1.0 and Human Druggable Genome siRNA Set V2.0; Qiagen) were arrayed in 384-well plates. To each well, 8 μl of serum-free RPMI medium (Invitrogen) containing 3.75% HiPerFect (Qiagen) was added. After 20-min incubation at room temperature, 30 μl of cell suspension containing 1,250 cells was added to give a final siRNA concentration of ∼20 nM. Cells were incubated at 37 °C and 5% CO_2_ for 72 h before infection with 3.5 × 10^4^ plaque-forming units (PFUs). At 18 h post infection, cells were fixed and nuclei stained with Hoechst. Infection protocol was set-up to reach ∼50% of infected cells at the end of the experiment, thus enabling the identification of both proviral and antiviral genes.

The numbers of CHIKV-infected and non-infected cells were determined using an automated microscope (Olympus Soft Imaging Solutions). Images were taken with 4,6-diamidino-2-phenylindole and GFP filter sets (AHF-Analysetechnik). Scan^R^ Analysis Software (Olympus Soft Imaging Solutions) was used to automatically identify and quantify GFP-positive cells (indicating infected cells) and cell nuclei. The number of automatically counted nuclei was further used to estimate the cytotoxic effects of specific siRNAs. The siRNA was classified as toxic if 1,000 or fewer nuclei were determined within one well of a 384-well plate. All multi-well pipetting steps were performed using a Biomek FX^P^ Laboratory Automation Workstation (Beckman Coulter). An siRNA library (Qiagen Hu_Genome 1.0 and Human Druggable Genome siRNA Set V2.0; Qiagen) containing four siRNAs per gene for the druggable genome^43^ and two siRNAs per gene for non-druggable and predicted genes was screened at least three times independently. The following siRNAs with the indicated target sequence were included in all screening plates as controls: siE1 5′-AACCGAUGAUAAGGCACGAAA-3′, siPLK1 5′-CACCAUAUGAAUUGUACAGAA-3′ and AllStars (Qiagen). For validation experiments, three siRNAs (Silencer Select) per gene were purchased from Ambion. The same transfection and screening conditions were used as in the primary screen.

### Microarray analysis

To allow exclusion of non-expressed genes from the hit identification process, microarray analyses of (i) AllStars-transfected and CHIKV-infected, (ii) AllStars-transfected and non-infected, (iii) non-transfected and CHIKV-infected, and (iv) non-transfected and non-infected HEK-293 cells were performed. Transfection was carried out by adding 4 × 10^5^ HEK-293 cells (in complete DMEM medium) to a mixture of serum-free RPMI medium, HiPerFect (Qiagen) and AllStars siRNAs (final siRNA concentration: 20 nM). Infection with CHIKV-GFP was performed 72 h post transfection at a multiplicity of infection (MOI) of 3. Cells were lysed 18 h p.i. using Trizol (Invitrogen), followed by isolation of RNA, which was subsequently subjected to microarray analysis. Correlation analysis revealed no significant influence of AllStars transfections (compared with non-transfected cells) on the global expression of cellular genes (correlation coefficient=0.992). Therefore, only the data set derived from transfected (CHIKV-infected and non-infected) HEK-293 cells was used for the removal of non-expressed genes (cutoff criterion: expression intensity>100), as described in data analysis below. Microarray experiments were performed as dual-colour hybridizations on Agilent 4 × 44 K human whole-genome catalogue arrays (Agilent-014850). To compensate for dye-specific effects, a dye-reversal colour swap was applied.

### Hit validation by CRISPR/Cas9-mediated gene knockout

Cas9-expressing HEK-293T and A549 cells were generated by transducing them first with lentiviruses based on the plasmid lentiCas9-Blast (Addgene number 52962), followed by selection with blasticidine for 10 days. Cells were then transduced with lentiviruses derived from the plasmid lentiGuide-Puro^44^ (Addgene number 52963), which leads to the expression of specific gRNAs) listed in Supplementary Data 4. Cas9-positive HEK-293T cells expressing individual gRNAs were generated by keeping cells under selection medium containing blasticidine (5 μg ml^−1^) and puromycin (2 μg ml^−1^) for an additional 10 days. A549 cells depleted for CLK1 were generated accordingly, but in this case single-cell clones were generated, which were kept under selection medium containing blasticidine (10 μg ml^−1^) and puromycin (2.5 μg ml^−1^) for an additional 10 days and subsequently analysed using the GeneArt Genomic Cleavage Detection Kit (Life Technologies) and the following oligonucleotides: CLK1_up: 5′-TGGAGTAGAGTGGCACGATG-3′; CLK1_down: 5′-GGATGCTTTAAGGCTTCTCTGA-3′ (uncropped gel presented in Supplementary Fig. 6). After the selection process, gRNA-expressing HEK-293T and A549 cells were infected with CHIKV-GFP for the periods of time and at MOIs as indicated.

### Western blotting and luminescence assay

For experiments in Fig. 3b,c, a combination of two siRNAs (Ambion) was used to silence FASN (s5030 and s5031), ACC (s882 and s883) or ACLY (s915 and s916). HeLa cells (at 40% confluency in 24-well plates) were transfected using Lipofectamine RNAiMAX reagent (Life Technologies), with 5 pmol of each siRNA (10 pmol in total) or 10 pmol of silencer negative control no. 2 siRNA (AM4613; Ambion). Knockdown effect was analysed 72 h post transfection by western blotting. Proteins were separated by 6% SDS–polyacrylamide gel electrophoresis, transferred to nitrocellulose membrane (GE Lifesciences) and detected using primary antibodies against ACC (clone EP687Y, from Abcam, ab45174, 1:1,000), ACLY (rabbit polyclonal from Abcam, ab137579, 1:1,000), FASN (rabbit polyclonal from Novus, NB400-114, 1:1,000) or β-actin (clone C4, Santa Cruz Biotechnology, sc-47778, 1:2,000). Appropriate horseradish peroxidase-conjugated secondary antibodies (LabAs Ltd) were used for visualization. Enhanced chemiluminescence reagent (GE Healthcare) was used to develop the blots (uncropped immunoblots presented in Supplementary Fig. 6). To perform the luciferase activity assay, siRNA-transfected HeLa cells were infected with Rluc- and Fluc-expressing CHIKV VRPs (MOI=0.01) at 72 h post transfection for 1 h. At 8 h p.i., cells were lysed using passive lysis buffer (Promega), and the relative activities of Rluc and Fluc were measured using a dual-luciferase detection kit (Promega). For experiments in Fig. 3f, specific FASN, ACC or ACLY chemical inhibitors were added from 1 h p.i. until the end of the experiment. The effects of drugs targeting the fatty acid biosynthesis pathway in HeLa cells were monitored in real time using the xCELLigence System (Roche) and corresponding electrode plates (E-plate). The system measures electrode impedance, which is given as cell index value. For the assay, HeLa cells were seeded in the wells of a E-plate and 18 h later growth medium pre-mixed with drugs was added to the wells to monitor the cell index change.

### Immunofluorescence

For indirect immunostaining, cells were fixed in 4% paraformaldehyde (PFA) and permeabilized with 0.1% Triton X-100 (Sigma). Antibodies were diluted in PBS containing 5% FCS. The following primary antibodies were used: rabbit anti-ACC (clone EP687Y, from Abcam, ab45174, 1:100), anti-ACLY (rabbit polyclonal from Abcam, ab137579, 1:100), anti-FASN (rabbit polyclonal from Novus, NB400-114, 1:100), mouse anti-dsRNA (clone J2, English and Scientific Consulting Kft, 1:200); incubation with primary antibodies was followed by treatment with secondary antibodies: anti-rabbit antibodies conjugated with Alexa Fluor 488 and anti-mouse antibodies conjugated with Alexa Fluor 568 (Life Technologies). Cells were observed using a Carl Zeiss LSM710 confocal microscope.

Immunostaining with the mouse anti-human-FLT4 (clone 9D9F9, Biolegend, 356201, 1:100) and rabbit anti-human-phospho-FLT4 (rabbit polyclonal from Cell applications, CY1115, 1:400; Supplementary Fig. 5b) was performed on 4% PFA-fixed cells, activated with −20 °C cold methanol (Sigma) for 20 min, and then permeabilized and stained in 0.05% saponin (Sigma) and 0.2% bovine serum albumin (BSA; Sigma) in PBS. Anti-mouse IgG antibodies conjugated with Alexa Fluor 647 and anti-rabbit antibodies conjugated with Alexa Fluor 488 were used as secondary antibodies. HeLa cells were transfected for 20 h (using the JetPRIME transfection reagent, Polyplus) with the pcDNA3.1-VEGFR-3 (overexpressing the wild-type form of FLT4) or with the pcDNA3.1-VEGFR-3-G857R (overexpressing a non-phosphorylatable mutant form of FLT4). The two plasmids were kindly provided by K. Alitalo, Helsinki).

### Chemical compound screening

For screening of compounds with CHIKV, HEK-293 cells were pretreated on 384-well plates with the various substances in eight different 1:3 dilutions 2 h before infection. Infection was performed using 3.5 × 10^4^ PFU CHIKV-GFP per well. At 18 h p.i., cells were fixed with 3.7% formaldehyde and the percentage of infected to non-infected cells quantified by staining nuclei with Hoechst, followed by automated microscopy and single object analysis. On the basis of the results of the eight different dilutions, dose–response curves were calculated using the R software package drc^45^ and the half-maximal inhibitory concentrationvalues of each compound determined. All multi-well pipetting steps were performed as described above.

To analyse primary infection of herpes simplex type 1 strain KOS (HSV-1), cowpox strain Brighton Red (CPXV) or adenovirus strain 5 (Ad5), HEK-293 cells were washed with PBS and incubated at 37 °C for 2 h with small molecules (73-0.033 μM in eight steps, alternatively for Bafilomycin 73-0.033 nM in eight steps) or an equivalent of DMSO under normal culture conditions. Cells were subsequently inoculated with GFP-expressing HSV-1, CPXV or Ad5 at MOI 5 (HSV-1) or MOI 2 (CPXV and Ad5) for 24 h before being fixed with 1% formaldehyde in PBS for 15 min. All virus strains expressed GFP, and the presence of GFP was determined by microscopic analysis performed as described previously^9^.

The same compound concentrations were used to test the antiviral efficacy against IAV/WSN/33 (IAV) viruses. Human lung epithelial A549 cells were pretreated with small molecules for 2 h and then inoculated with WSN at MOI 0.02. After 40 min, infection medium (DMEM supplemented with 0.2% BSA, 4 mM L-glutamine, antibiotics and TPCK-treated trypsin (1 μg ml^−1^), Sigma-Aldrich) was added, and the cells were incubated under normal culture conditions for 36 h. Virus load was determined by inoculating MDCK cells (seeded the day before) with the supernatants, followed by fluorescence microscopic analysis of infection. For this, MDCK cells were washed and inoculated with undiluted supernatant for 1 h. Virus was removed and MDCK cells were cultivated for 6 h under normal culture conditions with infection medium. Cells were stained for viral NP protein and nuclei, and the infection rate (that is, the rate of NP-positive cells) was determined as described previously^9^.

To exclude the possible cytotoxic effects, all compounds were additionally tested using the ‘Cell Proliferation Reagent WST-1' (Roche Diagnostics). HEK-293 cells were treated with the same concentrations of the various substances and 18 h later WST-1 was added. Readout was conducted using the EnVision Multilabel Reader (PerkinElmer).

### CLK1 inhibitor synthesis

The β-carboline-based CLK1 inhibitors (Supplementary Methods) were obtained as follows:^46^,^47^,^48^

*AnnH18* ((E)-7-methoxy-1-(2-phenylethen-1-yl)-9*H*-pyrido[3,4-b]indole) was prepared from harmine and benzaldehyde using the method of Li *et al*.^47^;

*AnnH14* (9-butyl-6-chloro-7-methoxy-1-methyl-9*H*-pyrido[3,4-b]indole) was prepared from 6-chloroharmine as follows: a solution of 53 mg (0.21 mmol) of 6-chloroharmine and 31 mg (0.27 mmol) of potassium *tert*-butanolate in 5 ml of anhydrous DMSO was stirred at 80 °C for 30 min, then 0.13 ml (1.1 mmol) of *n*-iodobutane was added slowly. After stirring at 80 °C for 2 h, the mixture was treated with aqueous ammonia (10%) and extracted with ethyl acetate. The organic layer was dried over sodium sulfate and evaporated. The residue was purified by flash column chromatography (silica; eluent: hexane/ethyl acetate/ethanol, 2:2:1) to give 35 mg (54%) of the target compound as a pale yellow solid, mp 133-137 °C. ^1^H-NMR (DMSO-D_6_, 400 MHz): *δ* (ppm)=8.34 (s, 1 H, 5-H), 8.19 (d, *J*=5.2 Hz, 1 H, 3-H), 7.94 (d, *J*=5.2 Hz, 1 H, 4-H), 7.37 (s, 1 H, 8-H), 4.58 (t, *J*=7.5 Hz, 2 H, 1′-H), 4.02 (s, 3 H, OCH_3_), 2.95 (s, 3 H, 1-CH_3_), 1.72-1.68 (m, 2 H, 2′-H), 1.37-1.31 (m, 2 H, 3′-H), 0.92 (t, *J*=7.4 Hz, 3 H, 4′-H). ^13^C-NMR (DMSO-D_6_, 400 MHz): *δ* (ppm)=150.7 (C-7), 136.7 (C-8a), 136.5 (C-1), 133.5 (C-3), 130.1 (C-9a), 123.2 (C-4a), 117.9 (C-5), 109.9 (C-4b), 109.6 (C-6), 108.1 (C-4), 89.6 (C-8), 52.1 (OCH_3_), 35.5 (C-1′), 28.0 (C-2′), 18.6 (1-CH_3_), 15.0 (C-3′), 9.3 (C-4′). HR-MS (EI): *m*/*z*=302.1193 (calculated for C_17_H_19_N_2_OCl:302.1186);

*AnnH80* (1-bromo-7,8-dichloro-9-(prop-2-yn-1-yl)-9*H*-pyrido[3,4-b]indole) was prepared in three steps from 7,8-dichloro-3,4-dihydro-1-oxo-9*H*-pyrido-[3,4-b]indole^49^ (see Supplementary Methods for experimental details and analytical data). Briefly, dehydrogenation of 7,8-dichloro-3,4-dihydro-1-oxo-9H-pyrido-[3,4-b]indole (A) was performed with 2,3-dichloro-5,6-dicyano-1,4-benzoquinone (DDQ) in tetrahydrofuran (THF) to give the 1-oxo-β-carboline (B; step 1). Subsequent bromination with phosphoryl bromide in anisole (step 2) gave the 1-bromo-β-carboline (C). This compound was converted to the target compound AnnH80 by deprotonation with sodium hydride, followed by N-alkylation with propargyl bromide (step 3).

### *In vitro* drug testing

The following drugs were used following solubilization in DMSO: tivozanib (Selleck, 43.96 mM stock), cerulenin (TEBU, 50 mM stock), KH-CB19 (R&D Systems, 10 mM stock), bafilomycin (50 μM stock), pimozide (10 mM stock), W7 (53 mM stock), TOFA (10 mM stock), BMS-303141 (25 mM stock) and (15:3)-anacardic acid (10 mM stock; all from Sigma). To determine the step of the viral cycle affected by chemical compounds, HEK-293T cells (seeded the day before on poly-L-lysine-coated plates) were either treated 2 h before or 2 h after cell infection with CHIKV C21 (MOI 40) and left until 8 h p.i. Viral infection was quantified by flow cytometry measurement of the % of capsid-expressing cells. Equal amounts of supernatants (3 μl) from the 2 h post treatment conditions were then measured for viral infectivity on Vero cells (final volume: 150 μl). The percentage of infected cells was measured by flow cytometry at the end of the experiment. For cell viability analysis, we took advantage of the high-precision measurement of the acquired volumes made by the MACSQuant VYB cytometers. Then, knowing the initial volume of cells, the acquired volume and the acquired number of events, we estimated the total number of cells per well. Cell staining for these experiments was performed in V-bottom 96-well plates (Nunc).

### Quantitative real-time PCR and flow cytometry

For vRNA quantification, vRNA was extracted from infection supernatants using the QIAamp Viral RNA Mini Kit (Qiagen), whereas total RNA from cells was extracted using the RNeasy Mini Kit (Qiagen). One-step quantitative real-time PCR reactions were conducted on an Applied Biosystems 7500 Fast system or a StepOnePlus Real-Time PCR System (Applied Biosystems) using the qPCR SyGreen 1-Step Lo-ROX or Hi-ROX mix (PCRBIOSYSTEMS) and the following primers: E1-C21_F (5′-ACGCAGTTGAGCGAAGCAC-3′), E1-C21_R (5′-CTGAAGACATTGGCCCCAC-3′), GAPDH_F (5′-GGTATCGTGGAAGGAAGGACTCATGAC-3′) and GAPDH_R (5′-ATGCCAGTGAGCTTCCGTTCAG-3′). vRNA present in infected cells was normalized on the GAPDH expression using the 2^−ΔΔCT^ method, whereas a standard curve derived from a viral stock of known titre was used to quantify vRNA in the supernatants. Cell viability in HeLa cells treated with the indicated drugs was measured using the CellTiter-Glo kit (Promega) and the Fluoroskan Ascent FL luminometer (Thermo Scientific).

Experiments in Supplementary Fig. 5d,e were performed using the HiPerFect transfection kit to transfect siRNA (Silencer Select Negative Control No. 2 siRNA, catalogue number 4390846, and s33489, s20629 and s31791—target sequences in Supplementary Data 1—at a final concentration of 5 nM) for 48 h before infection. Silencing efficiency was determined by quantitative PCR using the TaqMan RNA-to-Ct 1-Step Kit and the following TaqMan Gene Expression Assays (Life Technologies): Hs00197310_m1, Hs00367471_m1 and Hs00219525_m1. RNAs were extracted following the RNeasy Mini Kit (Qiagen) protocol with DNAse treatment.

Intracellular capsid (C) staining for flow cytometry was performed using a permeabilization buffer (PBS, 0.05% saponin and 0.2% BSA). We used a mouse monoclonal antibody (clone H3-18, 1:1,000) generated in-house and directly coupled with ATTO532 to reveal CHIKV capsid and a rabbit polyclonal serum recognizing the capsid protein (Ventoso I, Madrid, 1:1,000) to detect SINV. Flow cytometry acquisition was performed using a MACSQuant VYB system; FlowJo was used for the analysis. The percentage of infected cells was used as infection readout. Data were then normalized by setting the DMSO condition as an arbitrary value of 100.

Simultaneous detection of FLT4 (clone 9D9F9, Biolegend, 356201, 1:50) and P-FLT4 (rabbit polyclonal from Cell applications, CY1115, 1:400) was performed on CHIKV-GFP-infected cells (MOI: 10 for 18 h), fixed in 4% PFA, activated with −20 °C cold methanol for 20 min and stained in permeabilization buffer. Cells were acquired using an LSR-fortessa flow cytometer (BD). Anti-mouse IgG antibodies conjugated with Alexa Fluor 555 and anti-rabbit antibodies conjugated with Alexa Fluor 647 (both from Life Technologies) were used as secondary antibodies. We used cells stained with a mouse isotype control antibody (clone MOPC-21, Biolegend, 400101, 1:50) or with the anti-rabbit IgG secondary antibody only as labelling controls.

### *In vivo* experiments

All animal experiments were conducted according to project #2014-0019, which was approved by the Institut Pasteur Animal Ethics Committee on 17 October 2014. Two different mouse models of CHIKV infection were used for our studies: (i) the intradermal infection model of neonatal mice, characterized by CHIKV dissemination through the bloodstream, replication in different organs (liver, spleen, muscles and joints) and association with paralysis and death^32^; (ii) the footpad infection model, characterized by a rapid local replication of CHIKV at the site of infection and by the induction of inflammation and footpad swelling^36^,^37^,^38^. Both male and female mice were used for experiments in Fig. 6. Experiments in Fig. 7 were conducted on female mice. *Clk1* conditional knockout mice (C57BL/6NTac-Clk<tm1a(EUCOMM)Wtsi>/Ics) were purchased from ICS, France. Clk1 cKO mice were subsequently mated with Flp deleter (C57BL/6-Glipr2<GtIST12674H5TIGM>/J) and Cre deleter transgenic mice (B6.C-Tg(CMV-cre)1Cgn/J) to remove the βGeo cassette and the critical exons to generate *clk1* null alleles. Constitutive *clk1* knockout mice were further backcrossed into the C57BL/6J background (*N*=9, *F*=4). The wild-type C57BL/6J mice used for backcrossing were derived from the same unit and exposed to the same diet as the Clk1 null mice that were used as controls in those experiments. Mice were intradermally infected with CHIKV C21 (10^4^ PFU), weighed daily and killed at the appearance of paralysis.

For *in vivo* drug testing, C57BL/6J mice were purchased from Charles River Laboratories. Tivozanib-treated mice (0.5 mg kg^−1^, per os) were infected as described above, whereas pimozide (20 mg kg^−1^, via os)- and TOFA (25 mg kg^−1^, via intraperitoneal)-treated mice were subcutaneously infected with CHIKV C21 in the footpad (10^3^ PFU). Mouse health status and body weight were scored daily.

Drugs were handled as follows: for experiments shown in Fig. 6, tivozanib (Selleck) was first dissolved in DMSO (40 mg ml^−1^) and then diluted in 0.5% methylcellulose to a final concentration of 0.25 mg ml^−1^; pimozide (Sigma) was dissolved (25 mg ml^−1^) in *N*-methyl-2-pyrrolidone (NMP) and then diluted in PEG300 (5 mg ml^−1^); TOFA (Sigma) was dissolved in DMSO to a final concentration of 5 mg ml^−1^. For experiments shown in Fig. 7d,e, TOFA and pimozide were dissolved in DMSO (independently or in combination) to a final concentration of 3.64 and 4.55 mg ml^−1^, respectively, and dosed at 5.5 μl g^−1^. Littermate controls treated with appropriate vehicles were used for all experiments. CHIKV titrations from mouse explants were performed using the standard TCID50 dilution assay on Vero cells. Animal tissues were homogenized in DMEM (high glucose, GlutaMAX, pyruvate) at the final concentration of 0.1 g ml^−1^ using a Precellys 24 homogenizer (Bertin Technologies). In Fig. 7e, joint swelling was measured 4 days p.i. using a Schnelltaster caliper.

### Data analysis and reproducibility

For identification of primary hits, mainly the inhibitory effect on CHIKV, replication was taken into consideration. To reduce possible off-target or other unspecific effects and to maximize the robustness of the hit selection, two additional parameters were analysed: (i) non-expressed genes were excluded by determining constitutive or inducible expression via microarray profiling of non-infected and infected HEK-293 cells (7,561 genes were filtered out). (ii) Using the microscopic assay applied throughout the primary screen, toxic siRNAs, which reduced total cell numbers (<1,000 cells per well) on transfection were excluded. In this way, 3,274 siRNAs (targeting 2,635 different genes) were excluded from further analysis. The statistical analysis of the revised raw data was performed using cellHTS2 (ref. 15), an R-implemented software package for the analysis of cell-based high-throughput RNAi screen data. Raw data were normalized and centred by *Z*-score transformation. The medians of the centred and scaled values of at least three independent replicates were used for RSA analysis^16^. Primary hits were selected by using the cutoff criterion log *P*≤2.

The validation was performed based on the primary hit list, thereby focusing not only on proviral but also on antiviral factors. Although the validation was experimentally identical to the primary screening, the hit selection varied slightly: after filtering out 133 toxic siRNAs, the *Z*-scores were again determined using the cellHT2 software package. However, instead of using the RSA analysis, which has limitations in analysing data sets consisting of siRNAs that should theoretically all influence CHIKV replication, we computed the cSSMD based on the normalized per cent inhibition of individual siRNAs, by assessing the collective activity of multiple siRNAs relative to the control^17^. For the special case of only two siRNAs available, we used the simple method of moments estimate:


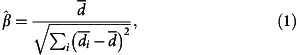


where 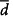 denotes the collective difference relative to control and 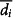 denotes the corresponding difference for the corresponding siRNA (*i*=1 and 2).

String analysis shown in Supplementary Fig. 1k was performed using only experimental data for prediction (confidence score≥0.5). Networks containing fewer than nine genes are not depicted. Molecular functions were arbitrarily assigned to the networks based on known functions.

The ingenuity analysis on the proviral factors, which is depicted in Fig. 1g, was performed using the ingenuity knowledge-base reference set (genes only), including direct and indirect relationships, and endogenous chemicals, and filtered for the ‘experimentally observed' confidence criterion. Enriched molecular functions were selected on the basis of their statistical significance (*P*≤0.01 was used as threshold). Statistically enriched gene ontology terms associated to less than two genes were removed. Co-localization analysis shown in Fig. 3e was performed on three-dimensional cell stacks using the intensity correlation analysis plugin for the WCIF Image J program. Statistical analyses in Figs 1, 3, 5, 6 and 7, Supplementary Fig. 1 and Supplementary Fig. 5 were performed using GraphPad prism. Networks in Fig. 1c and Supplementary Fig. 1k were drawn using the Cytoscape software. The STATA software (StataCorp) was used to calculate the Δ values for interaction effect on the geometric means of data shown in Fig. 7 following the two-factor design method^50^. No statistical method was used for the sample size determination and experiments were performed without randomization or blinding strategy.

## 

## Additional information

**Accession codes:** The microarray data have been deposited in NCBI's GENE expression Omnibus under accession code GSE69980.

**How to cite this article:** Karlas, A. *et al*. A human genome-wide loss-of-function screen identifies effective chikungunya antiviral drugs. *Nat. Commun.* 7:11320 doi: 10.1038/ncomms11320 (2016).

## Supplementary Material

Supplementary Information Supplementary Figures 1-6 and Supplementary Methods.

Supplementary Data 1 Best 4 siRNAs for proviral hits.

Supplementary Data 2 Best 4 siRNAs for antiviral hits.

Supplementary Data 3 Proviral genes in common between our and other RNAi screens for viral host factors.

Supplementary Data 4 Sequences of the gRNAs used for the CRISPR/Cas9 validation.

Supplementary Data 5 Association of the antiviral drugs with their proviral targets.

Supplementary Data 6 Principal biological functions for the hits of the antiviral drugs.

## Figures and Tables

**Table 1:** Inhibition of CHIKV replication by small molecule inhibitors.

Target protein or pathway	Drug	CHIKV IC_50_ (μM)	WST-1 IC_50_ (μM)	Therapeutic index
Fatty acid synthesis	TOFA	0.15	> 60*	NT
	Orlistat	0.82	8.67	10.57
	Cerulenin	3	7.57	2.53
vATPase	Bafilomycin	0.000334	0.003*	NT
CLK1	AnnH18	1.02	9.19	9.04
	AnnH14	1.55	10.55	6.79
	KH-CB19	3.36	10.93	3.26
KAT5	(15:3)-Anacardic acid	0.58	2.68	4.65
Calmodulin signalling	TAE684	0.15	2.07	14.28
	Pimozide	0.28	19.18	69.75
	Perphenazine	0.63	24.23	38.28
	6-Hydroxyflavone	2.6	> 60	NT
	Prenylamine	2.39	16.53	6.91
	Felodipine	3.24	12.08	3.73
FLT4	Alsterpaullone	0.002	44.38*	NT
	Pazopanib	0.13	1.32*	NT
	Axitinib	0.3	0.34*	NT
	Tivozanib	0.8	8.34*	NT
	Sorafenib	1.11	18.24	16.42
	Linifanib	2.15	> 60	NT

CHIKV, chikungunya virus; CLK1, CDC-like kinase 1; FLT4, fms-related tyrosine kinase 4; IC_50_, half-maximal inhibitory concentration; KAT5, K (lysine) acetyltransferase 5; NT, non-toxic; TOFA, 5-tetradecyloxy-2-furoic acid; vATPase, vacuolar-type H+ ATPase.

*Treatment induced <50% cell death.

List of selected compounds inhibiting CHIKV replication and their respective molecular targets or pathways. CHIKV infection and cell viability (WST-1) was measured in cells pretreated for 1 h with different dilutions of each drug (see Supplementary Fig. 4 for full data). Therapeutic index was calculated by dividing the WST-1 IC_50_ by the CHIKV IC_50_ value.

## References

[b1] Weaver S. C. & Lecuit M. Chikungunya virus and the global spread of a mosquito-borne disease. New Engl. J. Med. 372, 1231–1239 (2015). 25806915 10.1056/NEJMra1406035

[b2] Bekerman E. & Einav S. Infectious disease. Combating emerging viral threats. Science 348, 282–283 (2015). 25883340 10.1126/science.aaa3778 PMC4419706

[b3] De Clercq E. The design of drugs for HIV and HCV. Nat. Rev. Drug Discov. 6, 1001–1018 (2007). 18049474 10.1038/nrd2424

[b4] Scheel T. K. & Rice C. M. Understanding the hepatitis C virus life cycle paves the way for highly effective therapies. Nat. Med. 19, 837–849 (2013). 23836234 10.1038/nm.3248 PMC3984536

[b5] Lanford R. E. . Therapeutic silencing of microRNA-122 in primates with chronic hepatitis C virus infection. Science 327, 198–201 (2010). 19965718 10.1126/science.1178178 PMC3436126

[b6] Lin K. & Gallay P. Curing a viral infection by targeting the host: the example of cyclophilin inhibitors. Antiviral Res. 99, 68–77 (2013). 23578729 10.1016/j.antiviral.2013.03.020 PMC4332838

[b7] Garcia M. . Productive replication of Ebola virus is regulated by the c-Abl1 tyrosine kinase. Sci. Transl. Med. 4, 123ra124 (2012). 10.1126/scitranslmed.3003500 PMC4794994 22378924

[b8] Ashburn T. T. & Thor K. B. Drug repositioning: identifying and developing new uses for existing drugs. Nat. Rev. Drug Discov. 3, 673–683 (2004). 15286734 10.1038/nrd1468

[b9] Karlas A. . Genome-wide RNAi screen identifies human host factors crucial for influenza virus replication. Nature 463, 818–822 (2010). 20081832 10.1038/nature08760

[b10] Ooi Y. S., Stiles K. M., Liu C. Y., Taylor G. M. & Kielian M. Genome-wide RNAi screen identifies novel host proteins required for alphavirus entry. PLoS Pathog. 9, e1003835 (2013). 24367265 10.1371/journal.ppat.1003835 PMC3868536

[b11] Panda D. . Genome-wide RNAi screen identifies SEC61A and VCP as conserved regulators of Sindbis virus entry. Cell Rep. 5, 1737–1748 (2013). 24332855 10.1016/j.celrep.2013.11.028 PMC3920290

[b12] Varble A. . An in vivo RNAi screening approach to identify host determinants of virus replication. Cell Host Microbe 14, 346–356 (2013). 24034620 10.1016/j.chom.2013.08.007

[b13] Brass A. L. . Identification of host proteins required for HIV infection through a functional genomic screen. Science 319, 921–926 (2008). 18187620 10.1126/science.1152725

[b14] Konig R. . Human host factors required for influenza virus replication. Nature 463, 813–817 (2010). 20027183 10.1038/nature08699 PMC2862546

[b15] Boutros M., Bras L. P. & Huber W. Analysis of cell-based RNAi screens. Genome Biol. 7, R66 (2006). 16869968 10.1186/gb-2006-7-7-r66 PMC1779553

[b16] Konig R. . A probability-based approach for the analysis of large-scale RNAi screens. Nat. Methods 4, 847–849 (2007). 17828270 10.1038/nmeth1089

[b17] Zhang X. D. . cSSMD: assessing collective activity for addressing off-target effects in genome-scale RNA interference screens. Bioinformatics 27, 2775–2781 (2011). 21846737 10.1093/bioinformatics/btr474 PMC3202303

[b18] Tai A. W. . A functional genomic screen identifies cellular cofactors of hepatitis C virus replication. Cell Host Microbe 5, 298–307 (2009). 19286138 10.1016/j.chom.2009.02.001 PMC2756022

[b19] Li Q. . Integrative functional genomics of hepatitis C virus infection identifies host dependencies in complete viral replication cycle. PLoS Pathog. 10, e1004163 (2014). 24852294 10.1371/journal.ppat.1004163 PMC4095987

[b20] Sessions O. M. . Discovery of insect and human dengue virus host factors. Nature 458, 1047–1050 (2009). 19396146 10.1038/nature07967 PMC3462662

[b21] Krishnan M. N. . RNA interference screen for human genes associated with West Nile virus infection. Nature 455, 242–245 (2008). 18690214 10.1038/nature07207 PMC3136529

[b22] Konig R. . Global analysis of host-pathogen interactions that regulate early-stage HIV-1 replication. Cell 135, 49–60 (2008). 18854154 10.1016/j.cell.2008.07.032 PMC2628946

[b23] Sivan G. . Human genome-wide RNAi screen reveals a role for nuclear pore proteins in poxvirus morphogenesis. Proc. Natl Acad. Sci. USA 110, 3519–3524 (2013). 23401514 10.1073/pnas.1300708110 PMC3587217

[b24] Heaton N. S. . Dengue virus nonstructural protein 3 redistributes fatty acid synthase to sites of viral replication and increases cellular fatty acid synthesis. Proc. Natl Acad. Sci. USA 107, 17345–17350 (2010). 20855599 10.1073/pnas.1010811107 PMC2951450

[b25] Munger J. . Systems-level metabolic flux profiling identifies fatty acid synthesis as a target for antiviral therapy. Nat. Biotechnol. 26, 1179–1186 (2008). 18820684 10.1038/nbt.1500 PMC2825756

[b26] Varjak M. . Magnetic fractionation and proteomic dissection of cellular organelles occupied by the late replication complexes of Semliki Forest virus. J. Virol. 87, 10295–10312 (2013). 23864636 10.1128/JVI.01105-13 PMC3754020

[b27] Law V. . DrugBank 4.0: shedding new light on drug metabolism. Nucleic Acids Res. 42, D1091–D1097 (2014). 24203711 10.1093/nar/gkt1068 PMC3965102

[b28] Davis M. I. . Comprehensive analysis of kinase inhibitor selectivity. Nat. Biotechnol. 29, 1046–1051 (2011). 22037378 10.1038/nbt.1990

[b29] Gaulton A. . ChEMBL: a large-scale bioactivity database for drug discovery. Nucleic Acids Res. 40, D1100–D1107 (2012). 21948594 10.1093/nar/gkr777 PMC3245175

[b30] Sourisseau M. . Characterization of reemerging chikungunya virus. PLoS Pathog. 3, e89 (2007). 17604450 10.1371/journal.ppat.0030089 PMC1904475

[b31] Yasunaga A. . Genome-wide RNAi screen identifies broadly-acting host factors that inhibit arbovirus infection. PLoS Pathog. 10, e1003914 (2014). 24550726 10.1371/journal.ppat.1003914 PMC3923753

[b32] Couderc T. . A mouse model for Chikungunya: young age and inefficient type-I interferon signalling are risk factors for severe disease. PLoS Pathog. 4, e29 (2008). 18282093 10.1371/journal.ppat.0040029 PMC2242832

[b33] Bhargava P. & Robinson M. O. Development of second-generation VEGFR tyrosine kinase inhibitors: current status. Curr. Oncol. Rep. 13, 103–111 (2011). 21318618 10.1007/s11912-011-0154-3 PMC3047052

[b34] Nelson E. A. . The STAT5 inhibitor pimozide displays efficacy in models of acute myelogenous leukemia driven by FLT3 mutations. Genes Cancer 3, 503–511 (2012). 23264850 10.1177/1947601912466555 PMC3527989

[b35] Kang S., Roh Y. J. & Kim I. B. Antiangiogenic effects of tivozanib, an oral VEGF receptor tyrosine kinase inhibitor, on experimental choroidal neovascularization in mice. Exp. Eye Res. 112, 125–133 (2013). 23701975 10.1016/j.exer.2013.05.006

[b36] Ziegler S. A. . In vivo imaging of chikungunya virus in mice and Aedes mosquitoes using a Renilla luciferase clone. Vector Borne Zoonotic Dis. 11, 1471–1477 (2011). 21668347 10.1089/vbz.2011.0648 PMC3216093

[b37] Morrison T. E. . A mouse model of chikungunya virus-induced musculoskeletal inflammatory disease: evidence of arthritis, tenosynovitis, myositis, and persistence. Am. J. Pathol. 178, 32–40 (2011). 21224040 10.1016/j.ajpath.2010.11.018 PMC3069999

[b38] Teo T. H. . A pathogenic role for CD4+ T cells during Chikungunya virus infection in mice. J. Immunol. 190, 259–269 (2013). 23209328 10.4049/jimmunol.1202177

[b39] Vanlandingham D. L. . Development and characterization of a double subgenomic chikungunya virus infectious clone to express heterologous genes in Aedes aegypti mosquitoes. Insect Biochem. Mol. Biol. 35, 1162–1170 (2005). 16102421 10.1016/j.ibmb.2005.05.008

[b40] Couderc T. . Prophylaxis and therapy for Chikungunya virus infection. J. Infect. Dis. 200, 516–523 (2009). 19572805 10.1086/600381 PMC7109959

[b41] Glasker S. . Virus replicon particle based Chikungunya virus neutralization assay using Gaussia luciferase as readout. Virol. J. 10, 235 (2013). 23855906 10.1186/1743-422X-10-235 PMC3718613

[b42] Hoffmann E., Neumann G., Kawaoka Y., Hobom G. & Webster R. G. A DNA transfection system for generation of influenza A virus from eight plasmids. Proc. Natl Acad. Sci. USA 97, 6108–6113 (2000). 10801978 10.1073/pnas.100133697 PMC18566

[b43] Hopkins A. L. & Groom C. R. The druggable genome. Nat.Rev.Drug Discov. 1, 727–730 (2002). 12209152 10.1038/nrd892

[b44] Sanjana N. E., Shalem O. & Zhang F. Improved vectors and genome-wide libraries for CRISPR screening. Nat. Methods 11, 783–784 (2014). 25075903 10.1038/nmeth.3047 PMC4486245

[b45] Ritz C. S. & Streibig J. C. Bioassay analysis using R. J. Statist. Software 12, 1–22 (2005).

[b46] Ponce M. A. & Erra-Balsells R. Synthesis and isolation of bromo-beta-carbolines obtained by bromination of beta-carboline alkaloids. J. Heterocyclic Chem. 38, 1087–1096 (2001).

[b47] Li H.-Y., Koike K. & Ohmoto T. New alkaloids, picrasidines W, X and Y, from Picrasma quassioides and X-ray crystallographic analysis of picrasidine Q. Chem. Pharm. Bull. 41, 1807–1811 (1993).

[b48] Ponce M. A., Tarzi O. I. & Erra-Balsells R. Synthesis and isolation of chloro-beta-carbolines obtained by chlorination of beta-carboline alkaloids in solution and in solid state. J. Heterocyclic Chem. 40, 419–426 (2003).

[b49] Pohl B., Luchterhandt T. & Bracher F. Total syntheses of the chlorinated β-carboline alkaloids bauerine A, B, and C. Synth. Commun. 37, 1273–1280 (2007).

[b50] Krzywinski M. & Altman N. Points of significance: two-factor designs. Nat. Methods 11, 1187–1188 (2014). 25584374 10.1038/nmeth.3180

