# Supplementary material for: A human genome-wide loss-of-function screen identifies effective chikungunya antiviral drugs
Source: Nat Commun. 2016 May 12;7:11320. doi: 10.1038/ncomms11320 (PMC4865845; doi:10.1038/ncomms11320)
Supplement: Supplementary Information — Supplementary Figures 1-6 and Supplementary Methods. [file ncomms11320-s1.pdf]

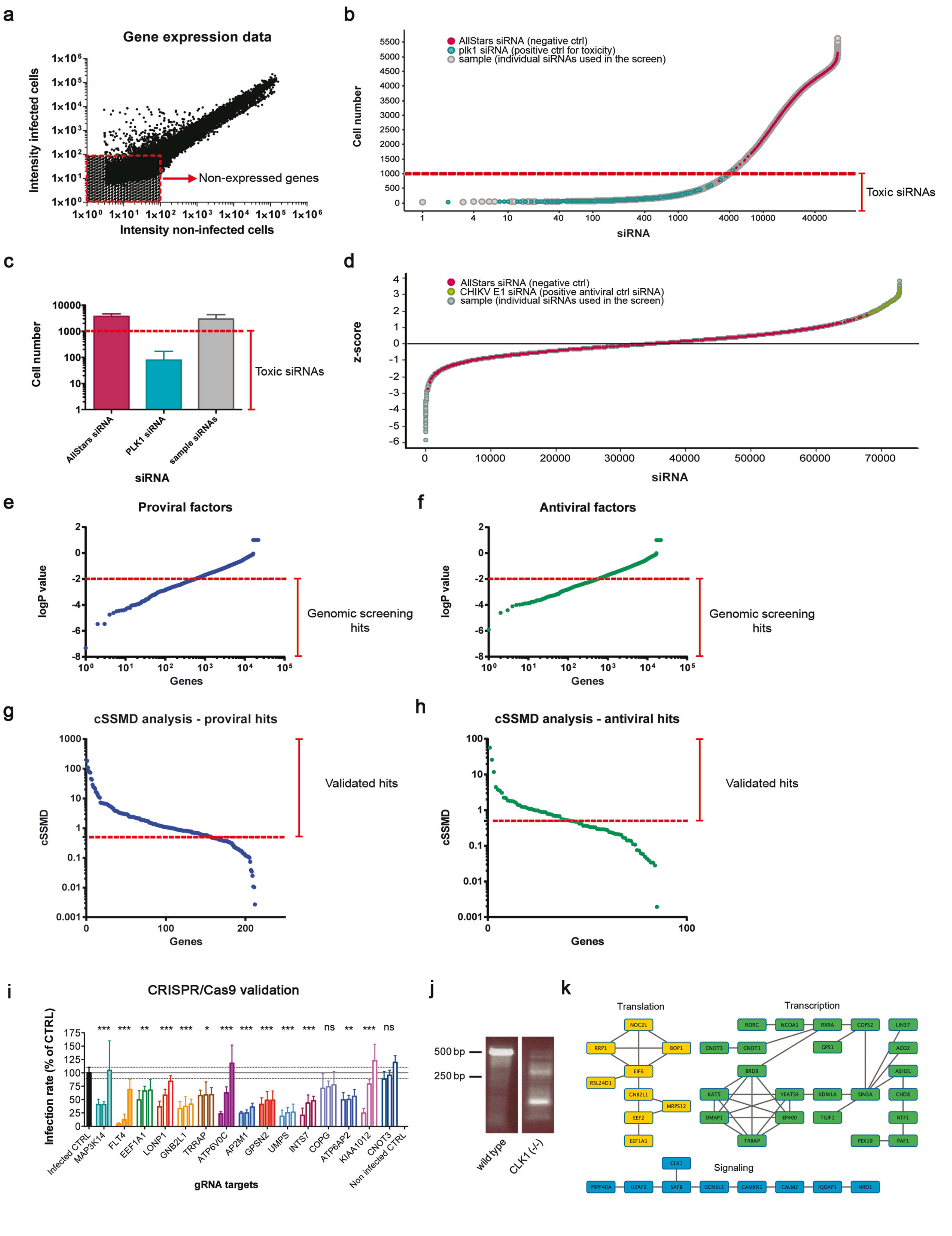

Supplementary Fig. 1: Quality controls and analysis of the genome-wide screening.

Filter criteria for the selection of CHIKV relevant hits. **a**, Genes with low expression levels (intensity values < 100) in infected and non-infected HEK-293 cells were excluded from further analysis. **b**, Toxic siRNAs that led to reduced cell numbers (< 1000) three days post-transfection were filtered out. siRNAs targeting the polo-like kinase 1 (plk-1) were included as positive control on every screening plate. **c,** Counts of cells transfected with indicated siRNAs during the

genome wide screen (n = 10016, 626 and 200320 for Allstars siRNA, PLK1 siRNA and sample siRNAs respectively). **d,** Distribution of all tested siRNAs and their ability to inhibit (positive Z-score) or enhance (negative Z-score) CHIKV replication. siRNAs targeting the viral E1 served as positive control. **e**, **f**, Selection of primary screening hits was based on redundant siRNA activity (RSA) analysis for proviral (**e**) and antiviral (**f**) factors. Genes with logP < -2 were classified as hits. **g, h,** Selected primary hits were validated using three additional siRNAs in the same screening assay. Proviral (**g**) and antiviral (**h**) factors were selected based on the calculated cSSMD values. Cut-off criteria for hit selection: cSSMD > 0.5. **i**, graph showing the mean values ± SD for the experiment described in Fig. 1d. The solid and the dotted black lines indicate the mean infection rate ± SD for the infected control (Cas9-expressing 293T cells). One-way Anova with Tukey's post test was used to calculate the significance of individual groups of gRNAs *versus* the infected control (*p < 0.05, **p < 0.01, ***p < 0.001, ^{ns} $p \geq 0.05$). **j,** CRIPSR/Cas-mediated gene knockout of CLK1 was confirmed using the GeneArt® Genomic Cleavage Detection Kit. **k**, Protein-protein interaction networks identified among the proviral genes by the string database (http://string-db.org/).

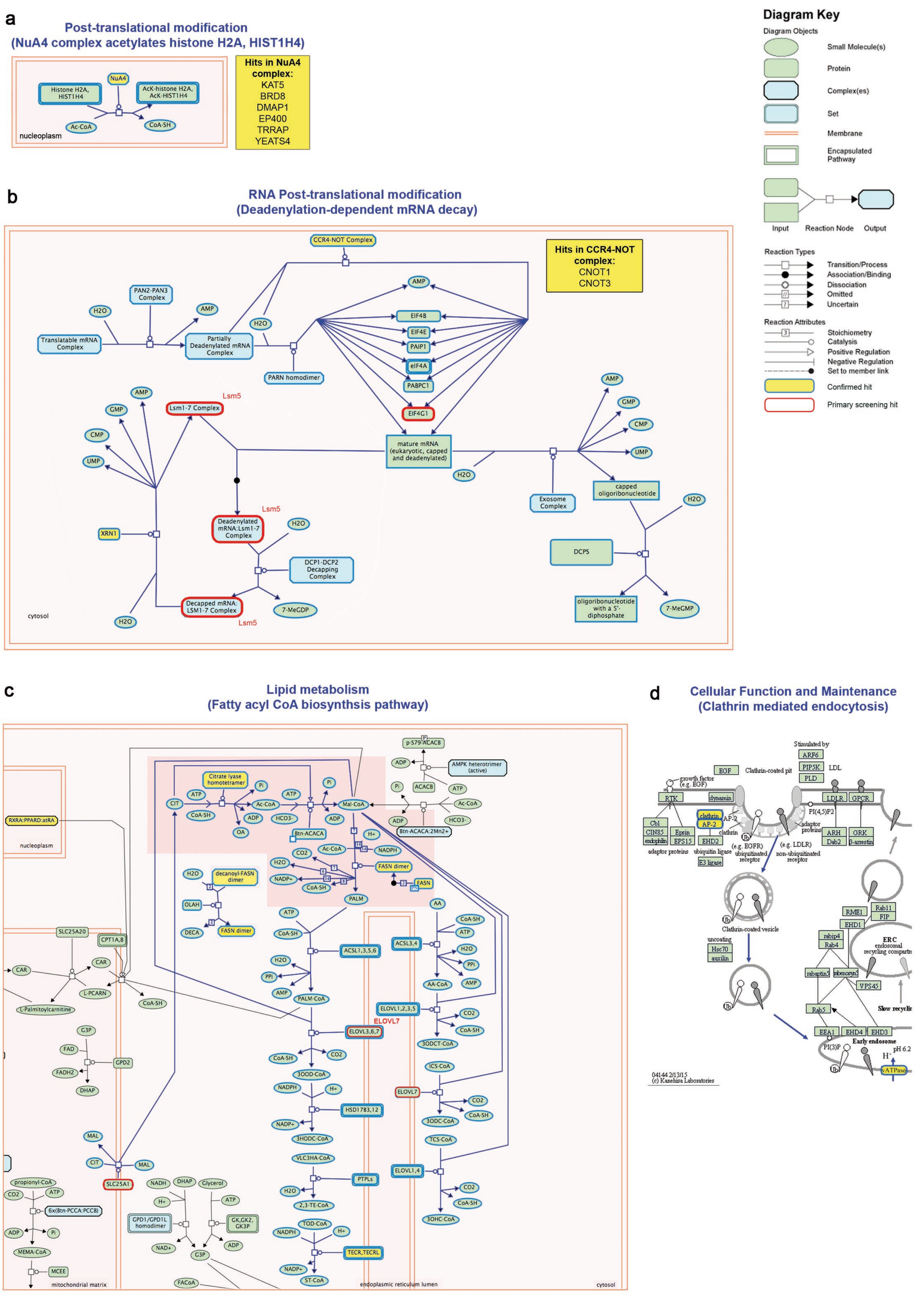

Supplementary Fig. 2: Illustration of some statistically enriched proviral pathways.

Graphical illustration of molecular functions that were statistically enriched in the proviral gene hit list (blue bars in Fig. 2b). Yellow boxes indicates proviral genes or gene lists present in a given protein complex. Red borders indicate hits

identified in the primary screen. Cartoons illustrating the biochemical pathways were downloaded from the reactome website (http://www.reactome.org, Cold Spring Harbor Laboratory, Ontario Institute for Cancer Research and the European Bioinformatics Institute) for **a**, **b** and **c** and from the KEGG pathway database (http://www.genome.jp/kegg/pathway.html, Kanehisa Laboratories) in **d** and modified to draw attention to CHIKV proviral factors. In *c* the pink background highlights the biochemical reaction involved in palmitate synthesis.

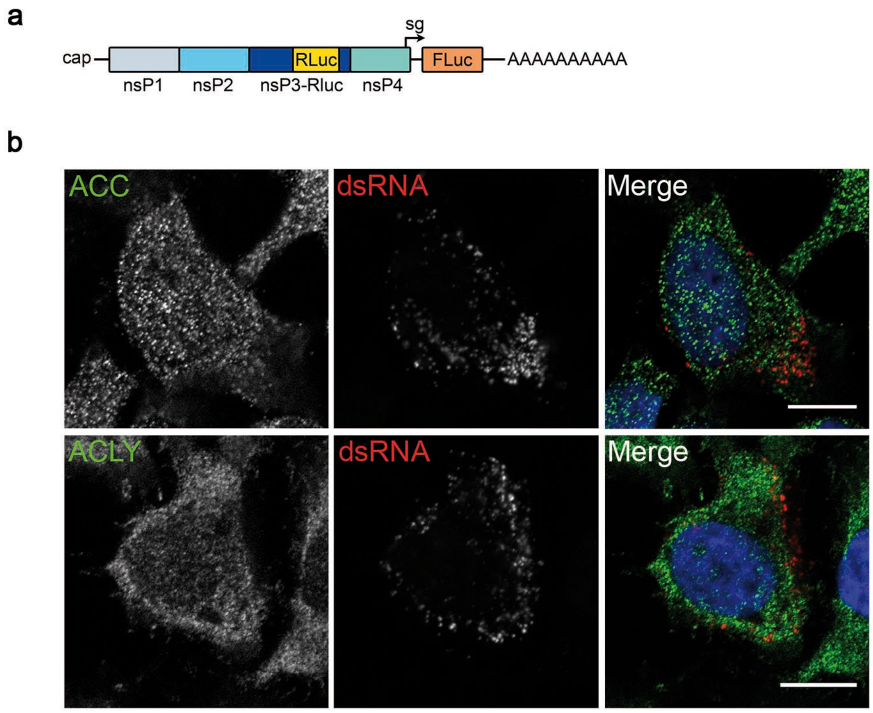

Supplementary Fig. 3: Fatty acid synthesis requirement for CHIKV lifecycle (Supplementary Information).

a, CHIKV replicon containing Renilla luciferase (RLuc) fused to the non-structural protein 3 (nsP3); Firefly luciferase (FLuc) expression is controlled by the subgenomic promoter. **b,** Confocal micrographs of HeLa cells treated as in Fig. 3d and labeled with the indicated antibodies and DAPI (blue). Scale Bars, 10 µm.

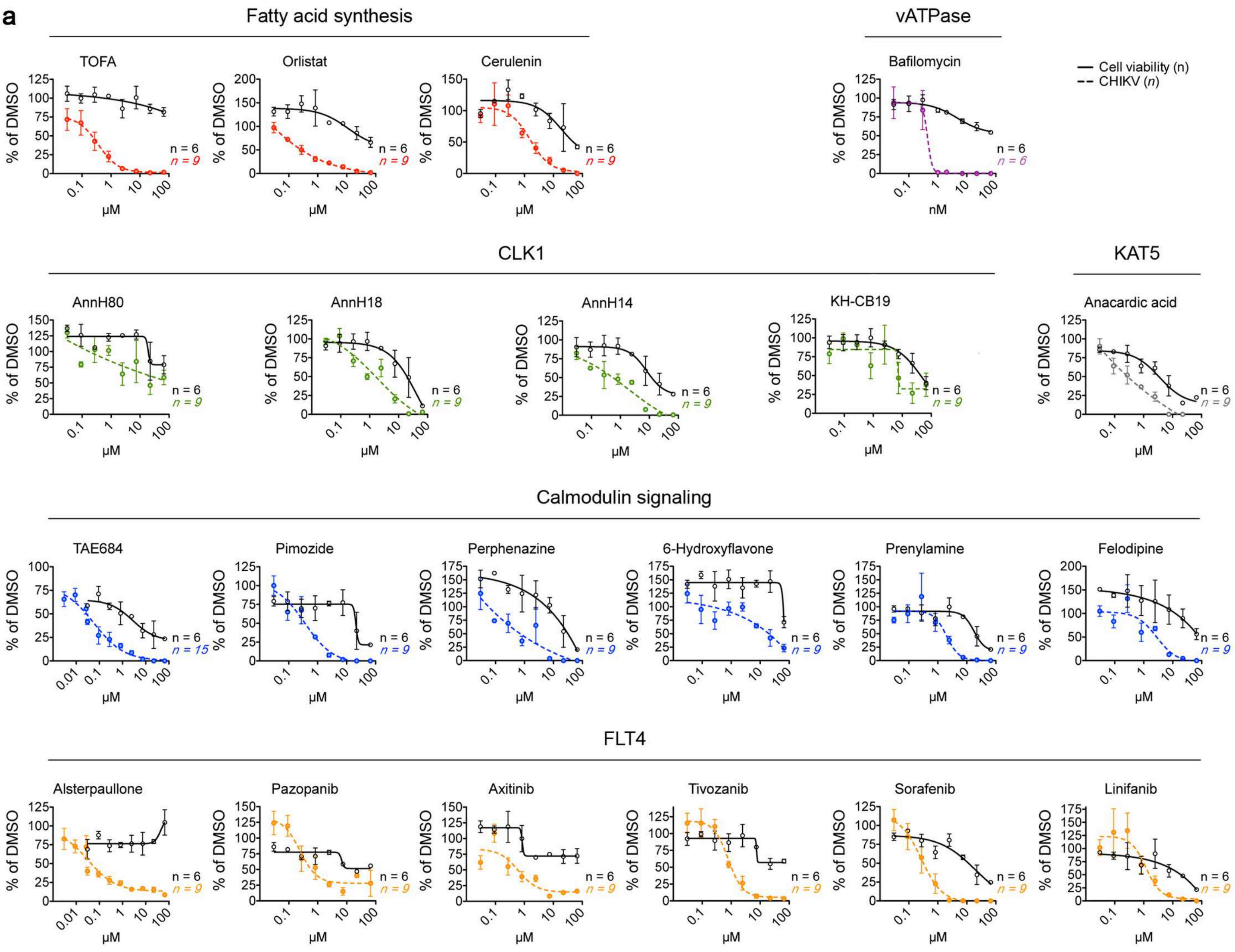

Supplementary Fig. 4: Dose response curves of the identified antiviral compounds.

a, Dose-dependent curves showing the inhibition of CHIKV replication induced by the indicated chemical inhibitors normalized to the vehicle control (DMSO, colored dashed lines). Cell viability data, based on the metabolic activity (WST-1 assay), at the indicated concentrations is shown in black. Mean values ± SEM derived from at least two independent experiments are shown.

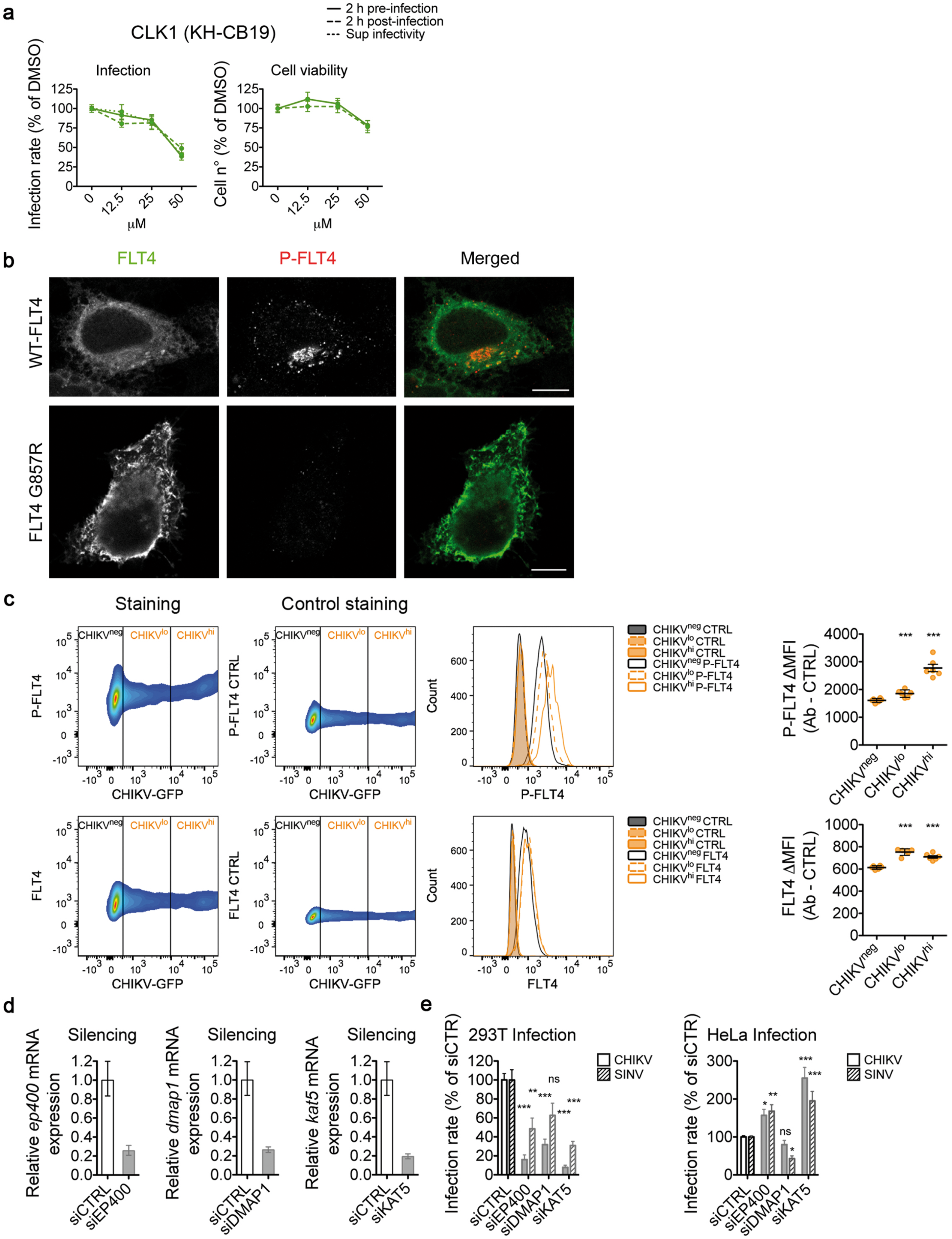

Supplementary Fig. 5: *In vitro* validation of selected chemical inhibitors and proviral factors (Supplementary Information).

a, Characterization of the viral cycle step affected by the CLK1 inhibitor KH-CB19 in HEK-293T cells (n = 9 for each dataset). **b,** Validation of an anti-FLT4 and of an anti-phospho-FLT4 (P-FLT4) antibody on HeLa cells overexpressing a wild type form or a non phosphorylatable mutant form of FLT4. Confocal sections showing the localization of FLT4

(green) and P-FLT4 (red) in the indicated cell populations are presented. Scale Bars, 10 µm. **c,** Flow cytometry plots of HEK-293T cells infected with CHIKV-GFP for 18 h, fixed and stained for FLT4 and P-FLT4. The difference between the median fluorescence intensity (MFI) of the indicated staining and the MFI of the control staining was calculated to quantify protein expression (n = 6). **d,** Silencing efficiency of siRNA specific for *ep400, kat5* and *dmap1,* measured by quantitative real-time PCR on total RNA extracts from HEK-293T cells at two days post-transfection. **e**, Quantification of CHIKV and SINV infection efficiency upon gene knockdown in the indicated cell lines was performed by flow cytometry, after labeling with CHIKV and SINV-CA antibody. Data represent % of siCTR (n = 9 for each dataset). Images from a representative experiment are shown in *b*. Except for *d,* where means ± SD of a representative experiment are depicted all other graphs represent means ± SEM of at least three independent experiments. Data were analyzed using one-way Anova with Tukey's post test (*p < 0.05, **p < 0.01, ***p < 0.001, ^{ns} p ≥ 0.05).

Full immunoblots (Fig. 3b)

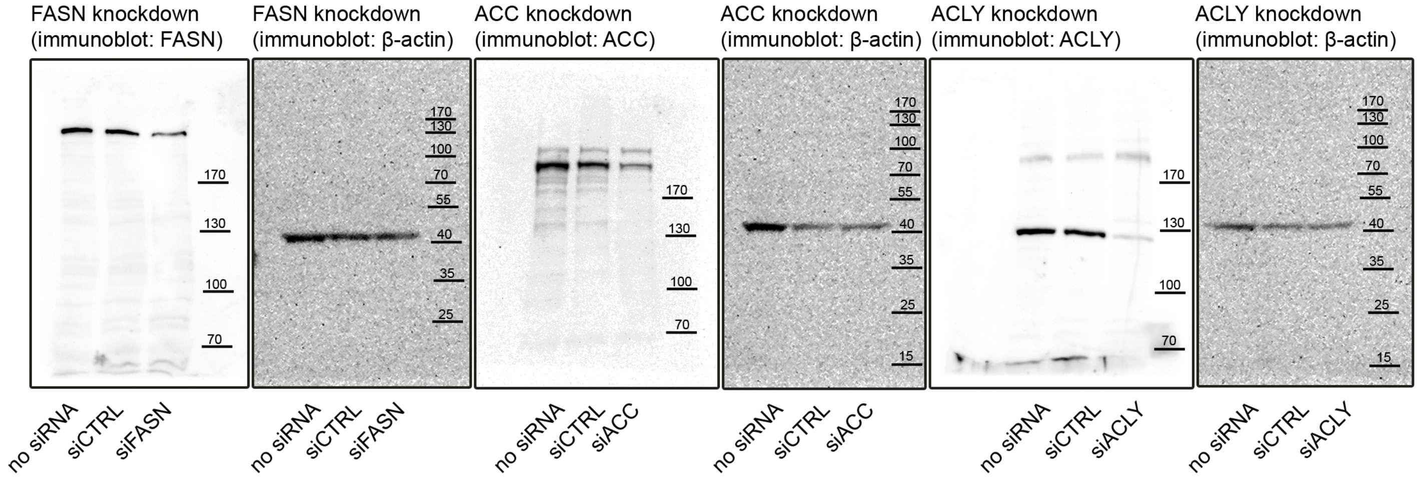

Full gel (Supplementary Fig. 1j)

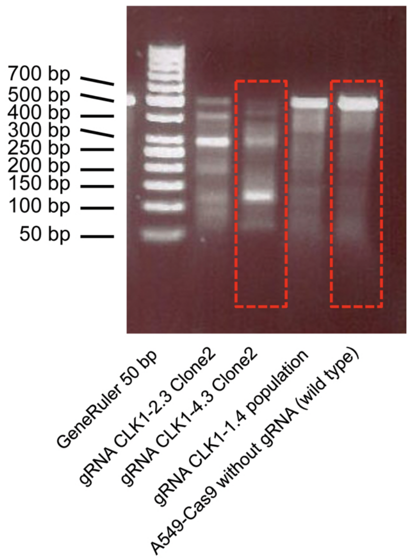

Supplementary Fig. 6: Pictures of uncropped immunoblots and gels

Supplementary Methods

Structure of newly developed CLK1 inhibitors AnnH14, AnnH18 and AnnH80. IC_{50} values of CLK1 inhibitors determined by capillary electrophoresis in a microfluidic chip system (LabChip3000, PerkinElmer) are indicated.

AnnH14 | AnnH18 | AnnH80

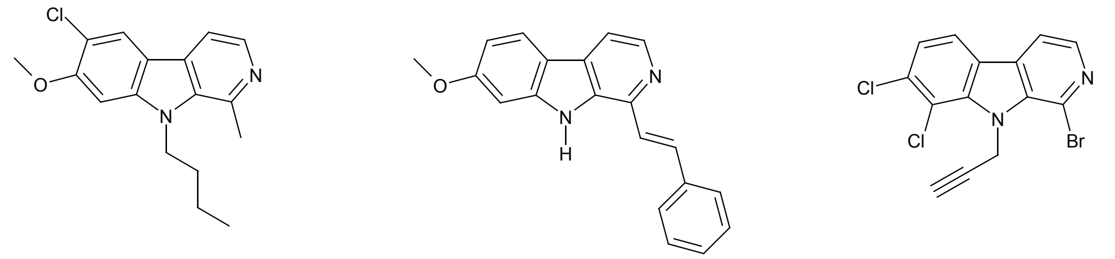

IC_{50} (CLK1) = 5.02 µM | IC_{50} (CLK1) = 0.575 µM | IC_{50} (CLK1) = 0.862 µM

Detailed description of the synthesis of AnnH80 and corresponding analytical data.

A | B | C | AnnH80

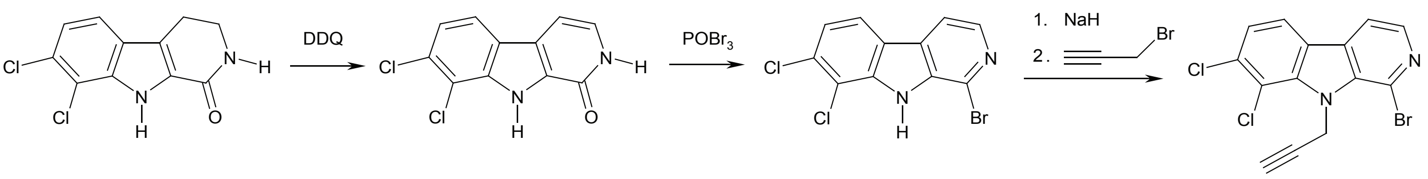

Step 1: A dispersion of 1.94 g (7.61 mmol) 6,7-dichloro-2,3,4,9-tetrahydro-1H-pyrido[3,4-b]indol-1-one (**A**) and 3.46 g (15 mmol) 2,3-dichloro-5,6-dicyano-1,4-benzoquinone (DDQ) in 120 mL tetrahydrofuran is refluxed under a nitrogen atmosphere for 15 h. After cooling to ambient temperature 55 mL ethyl acetate is added, and the mixture is extracted with 1M sodium hydroxide solution (4 x 100 mL). The combined aqueous layers are re-extracted with ethyl acetate (2 x 100 mL). The combined organic layers are concentrated, and the residue is washed with small amounts of warm ethyl acetate, methanol, and dichloromethane to give 1.27 g (67%) of 7,8-dichloro-2,9-dihydro-1H-pyrido[3,4-b]indol-1-one (**B**) as a very poorly soluble beige solid, mp 200 °C. ^{1}H-NMR (DMSO-D_6, 500 MHz): δ (ppm) = 12.52 (s, 1 H, 9-H), 11.58 (s, 1 H, 2-H), 8.07 (d, J = 8.2 Hz, 1 H, 5-H), 7.40 (d, J = 8.2 Hz, 1 H, 6-H), 7.19 (d, J = 6.4 Hz, 1 H, 3-H), 7.03 (d, J = 6.4 Hz, 1 H, 4-H). ^{13}C-NMR (DMSO-D_6, 400 MHz): δ (ppm) = 155.3 (C=O), 137.0 (C-8a), 129.3 (C-9a), 128.7 (C-8), 126.0 (C-3), 125.1 (C-4a), 122.4 (C-4b), 121.2 (C-6), 121.1 (C-5), 115.2 (C-7), 99.2 (C-4). HR-MS (EI): m/z = 251.9847 (calculated for $C_{11}H_6N_2Cl_2O$: 251.9857).

Step 2: A dispersion of 1.27 g (5.04 mmol) 7,8-dichloro-2,9-dihydro-1H-pyrido[3,4-b]indol-1-one (**B**) and 10 g (35 mmol) phosphoryl bromide ($POBr_3$) in 20 mL anisole is stirred at 120 °C for 4 h. After cooling to ambient temperature the mixture is treated with 130 mL saturated sodium carbonate solution and extracted with 4 x 200 mL ethyl acetate. The combined organic layers are dried over sodium sulfate and evaporated. The residue is purified by flash column chromatography (silica; eluent: hexane/ethyl acetate 1:1) to give 1.27 g (80%) of 1-bromo-7,8-dichloro-9Hpyrido[3,4-b]indole (**C**) as a beige solid, mp 200 °C. ^{1}H-NMR (DMSO-D_6, 500 MHz): δ (ppm) = 12.15 (s, 1 H, 9-H), 8.29 (d, J = 8.4 Hz, 1H, 5-H), 8.26 (d, J = 5.2 Hz, 1H, 4-H), 8.22 (d, J = 5.2 Hz, 1H, 3-H), 7.54 (d, J = 8.4 Hz, 1 H, 6-H). ^{13}C-NMR (DMSO-$D_{6,}$ 500 MHz): δ (ppm) = 140.0 (C-3), 139.2 (C-8a), 136.2 (C-4a), 131.8 (C-7), 130.2 (C-9a), 125.0 (C-1), 122.6 (C-6), 122.4 (C-5), 122.3 (C-4b), 115.9 (C-8), 115.8 (C-4). HR-MS (EI): m/z = 313.9005 (calculated for $C_{11}H_5N_2Cl_2Br$: 313.9013).

Step 3: A dispersion of 101 mg (0.32 mmol) 1-bromo-7,8-dichloro-9H-pyrido[3,4-b]indole (**C**) and 17 mg (0.43 mmol) sodium hydride (60% in mineral oil) in 2.5 mL N,N-dimethyl formamide is stirred at 40 °C for 30 min. Then 40 µL (0.37 mmol) propargyl bromide solution (80 % in toluene) is added, and the mixture is stirred at 40 °C for 3 h and at ambient temperature for 12 h. After careful addition of 10 mL saturated sodium carbonate solution and 5 mL water the mixture is extracted with ethyl acetate (3 x 20 mL). The combined organic layers are dried over magnesium sulfate and evaporated. The residue is purified by flash column chromatography (silica; eluent: hexane/ethyl acetate 3:2) to give 90 mg (79%) of the target compound 1-bromo-7,8-dichloro-9-(prop-2-yn-1-yl)-9H-pyrido[3,4-b]indole (**AnnH80**) as a light-brown solid, mp 190-193 °C. ^{1}H-NMR ($CDCl_3$, 500 MHz): δ (ppm) = 8.30 (d, J = 5.0 Hz, 1 H, 3-H), 7.93 (d, J = 8.3 Hz, 1 H, 5-H), 7.87 (d, J = 5.0 Hz, 1 H, 4-H), 7.48 (d, J = 8.3 Hz, 1 H, 6-H), 6.07 (d, J = 2.4 Hz, 2 H, 1′-H), 2.33 (t, J = 2.4 Hz, 1 H, 3′-H). ^{13}C-NMR ($CDCl_3$, 500 MHz): δ (ppm) = 140.7 (C-3), 138.8 (C-8a), 135.5 (C-9a), 135.0 (C-7), 132.3 (C-4a), 124.0 (C-1), 123.8 (C-6), 123.1 (C-4b), 120.0 (C-5), 117.0 (C-8), 114.0 (C-4), 79.0 (C-2′), 74.1 (C-3′), 36.1 (C-1′). HR-MS (ESI): m/z = 352.9243 (calculated for $C_{14}H_8N_2BrCl_2$: 352.9247).
